# Virtual Electroencephalogram Acquisition: A Review on Electroencephalogram Generative Methods

**DOI:** 10.3390/s25103178

**Published:** 2025-05-18

**Authors:** Zhishui You, Yuzhu Guo, Xiulei Zhang, Yifan Zhao

**Affiliations:** 1 Sino-French Engineer School, Beihang University, Beijing 100080, China; 18241094@buaa.edu.cn; 2 School of Automation Science and Electrical Engineering, Beihang University, Beijing 100080, China; 09494@buaa.edu.cn; 3 Boardware-Barco-Beihang BAIoT Brain Computer Intelligence Joint Laboratory, Beijing 100191, China; 4 Data Science Centre for Life-Cycle Engineering and Management, Cranfield University, Bedford MK43 0AL, UK; yifan.zhao@cranfield.ac.uk

**Keywords:** EEG generative models, variational autoencoders, generative adversarial networks, diffusion models, brain-computer interfaces

## Abstract

Driven by the remarkable capabilities of machine learning, brain–computer interfaces (BCIs) are carving out an ever-expanding range of applications across a multitude of diverse fields. Notably, electroencephalogram (EEG) signals have risen to prominence as the most prevalently utilized signals within BCIs, owing to their non-invasive essence, exceptional portability, cost-effectiveness, and high temporal resolution. However, despite the significant strides made, the paucity of EEG data has emerged as the main bottleneck, preventing generalization of decoding algorithms. Taking inspiration from the resounding success of generative models in computer vision and natural language processing arenas, the generation of synthetic EEG data from limited recorded samples has recently garnered burgeoning attention. This paper undertakes a comprehensive and thorough review of the techniques and methodologies underpinning the generative models of the general EEG, namely the variational autoencoder (VAE), the generative adversarial network (GAN), and the diffusion model. Special emphasis is placed on their practical utility in augmenting EEG data. The structural designs and performance metrics of the different generative approaches in various application domains have been meticulously dissected and discussed. A comparative analysis of the strengths and weaknesses of each existing model has been carried out, and prospective avenues for future enhancement and refinement have been put forward.

## 1. Introduction

Electroencephalography (EEG) is a non-invasive approach of measuring the brain’s electrical fields. It records the voltage potentials generated by the flow of electric current in and around neurons by means of electrodes placed on the scalp. EEG research has a history of nearly a century, during which it has accumulated a wealth of experience in various application areas. It not only has an important foundation in clinical diagnosis but also plays a role in more modern brain-triggered neurorehabilitation treatments [1]. It aids in diagnosing various neurological disorders, such as epilepsy, tumors, cerebrovascular lesions, depression, and trauma-related issues. Furthermore, as a true neuroimaging method, EEG has seen expanded applications in translational neuroscience and computational neuroscience in recent years [1].

BCI (brain–computer interface) is a technology that facilitates interaction between humans and computers by capturing and interpreting the brain’s electrical signals. This activity can be in the form of EEG or other electrophysiological recordings, such as Magnetoencephalography (MEG), Electrocorticography (ECoG), functional Magnetic Resonance Imaging (fMRI), and functional Near-Infrared Spectroscopy (fNIRs) [2]. However, compared to other recording methods, the cost of obtaining EEG is relatively low, making it more accessible [2]. The concept of BCIs, introduced in the 1970s by Jacques Vidal [3], has been further developed thanks to advancements in computer technology, machine learning, and neuroscience.

With the advancement of deep learning, substantial data are required to train large models with strong generalization capabilities. Generative models are a main solution of data augmentation. In the past decades, generative models for augmenting EEG data have achieved significant success. However, there is currently a lack of reviews comparing the applications of generative models in EEG data augmentation. Existing reviews only focus on a single type of generative model (e.g., GAN [4]). Therefore, this review aims to provide a broader overview, primarily examining EEG generative models based on VAE, GAN, and diffusion models. For each type of model, we provide a detailed introduction to the principles, model architecture, datasets used, and experimental results. At the end of each section, we discuss the advantages and limitations of each model.

By organizing and analyzing the existing studies on generating EEG signals based on generative models, several review studies have been conducted in this field, as shown in Table 1. Previous work on EEG-based data generation has comprehensively analyzed the improvement of generative models as well as the design of the experimental process, including experimental design methods, data preprocessing methods, feature extraction methods, and the effect of using generative models on performance. However, most of these works only explored a single generative model or a single task domain, neglecting the overall perspective of the EEG generation domain. We have conducted an all-encompassing review of this topic, and in this paper, we synthesize representative research results on generative models used for various EEG tasks in recent years.

The remainder of this review is organized as follows: In Section 2, we discuss the importance of data augmentation for EEG and review the most widely used methods. In Section 3, we discuss the literature search criteria and methodology. In Section 4, we introduce the theoretical foundations of three mainstream generative models and describe the evaluation metrics used in this review. In Section 5, Section 6 and Section 7, we review the applications of VAEs, GANs, and diffusion models in EEG generation and summarize their respective advantages and disadvantages, respectively. In Section 8, we conclude this paper and offer our views on future directions for development.

## 2. Data Augmentation

The rapid development of BCI based on deep learning in recent years has been remarkable, but breakthroughs in deep learning often depend on large datasets, which determine the final performance of the model. Unfortunately, currently, there are very few open-source EEG datasets available. This scarcity is mainly because annotating EEG data requires specific expertise, and the process is time-consuming and the results can vary depending on the annotator. Additionally, due to the lack of data, deep learning models find it difficult to generalize to unseen research subjects. Without appropriate regularization, the scarcity of data can severely hinder model generalization and lead to overfitting [10,11]. In addition to the well-known challenges associated with data annotation and limited availability, it is essential to acknowledge that EEG signal variability is substantially affected by the underlying neurological pathology. Distinct disorders often give rise to specific alterations in brain activity, which are reflected in the EEG signal morphology and spectral characteristics. When combined with inherent inter-subject variability, these pathological differences significantly increase the complexity and heterogeneity of EEG datasets [12]. Due to the inherently high-dimensional nature of EEG signals (characterized by multiple electrode channels, long temporal sequences, and substantial inter-subject variability), training robust and generalizable models remains highly challenging. These complexities often lead to increased computational burden, elevated risk of overfitting, and limited model scalability under constrained data conditions. Moreover, the spatial and temporal correlations across channels further complicate feature extraction and classification [13]. Additionally, EEG signals are highly susceptible to various forms of artifacts and environmental noise, including ocular and muscular movements, which can significantly distort the underlying neural patterns. Without adequate preprocessing and filtering strategies, such artifacts may introduce substantial variability and degrade the reliability of feature extraction and classification.

To mitigate these challenges, data augmentation (DA) has emerged as a necessary strategy to expand the diversity and quantity of training data. EEG signals in real-world applications are influenced by multiple factors, including pathological heterogeneity (that is, differences in characteristics across various disorders), inter-channel coupling, artifacts and noise, and physiological variability both between subjects and within a subject. Therefore, augmentation methods that are carefully designed, such as injecting jitter in the time domain, randomly dropping individual channels, or perturbing spectral components, can expose the model during training to a wide range of possible signal distortions and thus enable robust extraction of critical features in practical settings. In the field of computer vision, researchers enhance image datasets through flipping, rotating, scaling, cropping, shifting, adding Gaussian noise, etc. In recent years, researchers have also utilized deep learning-based generative models to generate additional samples from existing ones.

Inspired by the achievements of data augmentation in the field of computer vision, DA in BCI is also divided into two categories: methods based on geometric manipulation and methods based on generative models. Geometric manipulation is one of the simplest and most effective DA methods. For EEG data, geometric manipulations include time domain enhancement, frequency domain enhancement, and spatial domain enhancement.

In the time domain, Schirrmeister et al. generated additional data by sliding and cropping on longer EEG signals using shorter time windows [14]. Yang et al. added Gaussian noise to motor imagery EEG samples to increase data diversity [15]. Lotte et al. enhanced datasets by splitting EEG signals into multiple time segments and then recombining different segments [16]. Mohsenvand et al. randomly selected a portion of the signal and set it to zero to enhance the model’s ability to learn the differences between different augmented samples [17]. Rommel et al. augmented data by inverting the temporal order in the EEG signals [18]. In this way, the model may learn to recognize samples that remained unchanged even when the temporal sequence had been reversed, thereby enhancing its ability to generalize to the data. The team also performed data augmentation by randomly changing the polarity of the signals in all channels, an operation that does not change the semantic information of the data and also helps the model to recognize and differentiate between different instances of augmentation from the same data point as it learns.

In the frequency domain, Mohsenvand et al. performed a Fourier transform on the EEG signal to obtain its frequency components, then randomly changed the phase of these components while keeping the amplitude constant, and finally converted these modified frequency components back to the time domain through an inverse Fourier transform to achieve data augmentation [17]. Schwabedal et al. generated new data sequences by changing the Fourier phase of signals to address class imbalance [19]. Cheng et al. selected a specific frequency band and filtered out all frequency components within this band from the signal to simulate frequency-specific interference or signal loss [20].

In the spatial domain, Zhang et al. rotated, translated, and added random noise to spectrograms based on short-time Fourier transform (STFT) for data augmentation [21]. Shovon et al. performed DA on motor imagery EEG spectrograms by rotating, flipping, scaling, and brightening them [22]. Sakai et al. established a dual strategy to amplify all time data and near-peak data for data augmentation [23]. Deiss et al. enhanced EEG datasets by flipping the electrodes on the left and right sides of the brain [24]. Saeed et al. first randomly permuted channels and then randomly sampled a binary mask to completely discard some channels to increase the model’s robustness in handling missing and disordered channels [25].

Data augmentation methods based on generative models refer to the use of additional deep learning-based generative models to synthesize training samples. Among the deep generative models that have recently been successful, VAEs (Variational Autoencoders), GANs (Generative Adversarial Networks), and diffusion models have demonstrated their practical capabilities with a solid theoretical foundation. This paper systematically summarizes the applications of these three generative models in generating EEG data.

## 3. Search Method

**Figure 1:**
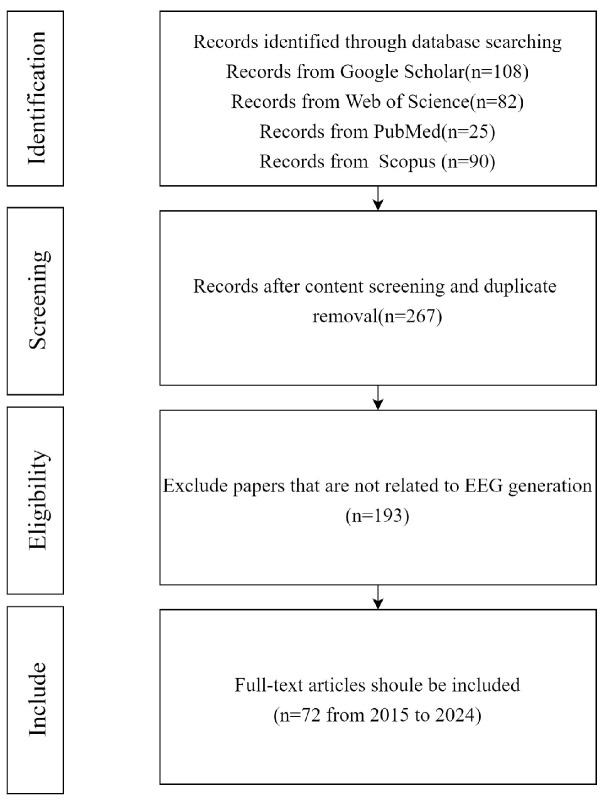
Selection criteria.

The main purpose of this paper is to study various generative models applied in different EEG-related tasks, emphasizing how these models generate EEG and the performance results they achieve. A literature review was conducted, as shown in Figure 1, across four databases including Google Scholar, Web of Science, PubMed, and Scopus, using the following group of keywords: (“Data augmentation” OR “Generate” OR “Synthesize”) AND (“Generative models” OR “GAN” OR “VAE” OR “Diffusion”) AND (“EEG” OR “Electroencephalography”). The initial search yielded 305 matching results in the databases, with publications ranging from 2015 to 2024, including 38 duplicate articles. After manually screening the remaining 267 papers, 195 were found to be irrelevant for this review (for example, the papers did not emphasize the role of generative models in EEG generation but focused more on the analysis of EEG signals). Thus, we ultimately selected 72 papers. After identifying papers that met the criteria based on our selection criteria, we extracted key information from each paper, including the following: authors, year of publication, main purpose of the article, dataset used, type of model, evaluation metrics used, and final experimental results.

## 4. Basic Concepts

### 4.1. VAE

**Figure 2:**
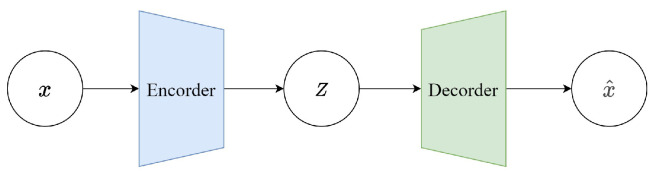
VAE architecture.

Variational Autoencoders (VAEs) were proposed by Kingma et al. in 2013 as a generative network structure based on Variational Bayes (VB) inference [26]. Unlike traditional autoencoders that describe the latent space numerically, it characterizes observations of the latent space probabilistically. This means that the output of the decoder is not the direct values of *x*, but the parameters of the probability distribution of *x*. Therefore, it has tremendous application value in data generation. Figure 2 illustrates a simplified schematic of a VAE. The model consists of two parts: the encoder and the decoder. The encoder’s function is to map the input data *x* to a representation in the latent space *z*. In a VAE, the encoder does not output a fixed *z* but instead outputs two parameters: the mean μ and the variance $\sigma^{2}$ (in practice, the logarithm of the variance is often used for numerical stability). These parameters define a probability distribution, typically assumed to be Gaussian, meaning *z* is sampled from N(μ,$\sigma^{2}$*I*). This probabilistic output is what differentiates a VAE from a traditional autoencoder. The decoder’s function is to map the latent representation *z* back to the data space, essentially reconstructing the input data *x*. In a VAE, the decoder learns the conditional probability $p_{\theta }$(x∣z), which is the probability of generating data point *x* given the latent representation *z*. The output of the decoder can be viewed as a “reconstruction” of the original input data.

During the training of a VAE, there are two goals: ensuring that the distribution of the latent representations output by the encoder is as close as possible to the prior distribution (usually a standard normal distribution) and ensuring that the data reconstructed from the latent representation are as similar as possible to the original data. To achieve these goals, the loss function of a VAE consists of two parts: reconstruction loss and Kullback–Leibler (KL) divergence. Reconstruction loss is typically the difference between the input data and the reconstructed data, like mean squared error loss or cross-entropy loss. KL (Kullback–Leibler) divergence measures the divergence between the distribution of the latent representations output by the encoder and the prior distribution. This metric encourages the encoder’s output distribution of *z* not to stray too far, thereby avoiding “overfitting” to specific features of the training data:

(1) $$\begin{array}{cc} \mathrm{KL}\left(q_{\phi }\left(z|x_{i}\right)\;∥\;p\left(z\right)\right)\; & =\mathrm{KL}\left(N\left(\mu \left(x_{i}\right),\Sigma \left(x_{i}\right)\right)\;∥\;N\left(0,I\right)\right)\\ \; & =\frac{1}{2}\left(\mathrm{tr}\;\Sigma \left(x_{i}\right)+\mu {\left(x_{i}\right)}^{T}\mu \left(x_{i}\right)-k-\log\det\Sigma \left(x_{i}\right)\right)\\ \; & =\frac{1}{2}\sum_{d=1}^{k}\left(\sigma_{d}^{2}\left(x_{i}\right)+\mu_{d}^{2}\left(x_{i}\right)-1-\log\sigma_{d}^{2}\left(x_{i}\right)\right). \end{array}$$

In this equation, the term $\mathrm{KL}\left(q_{\phi }\left(z|x_{i}\right)\;∥\;p\left(z\right)\right)$ quantifies the divergence between the encoder’s approximate posterior $q_{\phi }\left(z|x_{i}\right)$ and the prior p(z). Here, $x_{i}\in R^{d}$ denotes the *i*-th input sample, and $z\in R^{k}$ is its latent representation of dimension *k*. The encoder defines $q_{\phi }\left(z|x_{i}\right)=N\left(\mu \left(x_{i}\right),\Sigma \left(x_{i}\right)\right)$, where $\mu \left(x_{i}\right)\in R^{k}$ is the mean vector and $\Sigma \left(x_{i}\right)\in R^{k\times k}$ is the covariance matrix (typically diagonal, with entries $\sigma_{1}^{2}\left(x_{i}\right),\ldots ,\sigma_{k}^{2}\left(x_{i}\right)$). The prior p(z) is chosen as the standard normal N(0,I). In the closed-form KL expression, $\mathrm{tr}(\Sigma \left(x_{i}\right))$ is the sum of the diagonal variances, $\det\Sigma (x_{i})$ its determinant, and log denotes the natural logarithm. Minimizing this KL divergence encourages the learned posterior to stay close to the fixed prior.

The final goal of VAE is as follows:

(2) $$\begin{array}{cc} \max\;ELBO & =\sum_{i}ELBO_{i}\\ \; & =\sum_{i}\sum_{j}\left[{\left(x_{i}-x_{i}^{\prime }\right)}^{2}+\frac{1}{2}\Sigma \left(x_{i}\right)+\mu^{2}\left(x_{i}\right)-1\right.\\ \; & \;\left.-\log\Sigma (x_{i})\right], \end{array}$$

where $x_{i}\in R^{d}$ denotes the input data sample, and $x_{i}^{\prime }$ is the corresponding reconstruction generated by the decoder. The index j=1,2,…,d represents the feature dimensions of each input vector. The reconstruction loss $\sum_{j}{\left(x_{i}-x_{i}^{\prime }\right)}^{2}$ ensures that the reconstructed data are as close as possible to the original input. The terms $\mu (x_{i})$ and $\Sigma (x_{i})$ are the mean and (diagonal) covariance output by the encoder for sample $x_{i}$, where $\mu \left(x_{i}\right)\in R^{k}$ and $\Sigma \left(x_{i}\right)\in R^{k\times k}$, with *k* being the dimensionality of the latent variable *z*. The notation $\mu^{2}\left(x_{i}\right)$ denotes the squared $L^{2}-\mathrm{norm}$ of the mean vector, defined as $\sum_{d=1}^{k}\mu_{d}^{2}\left(x_{i}\right)$, and $\log\Sigma (x_{i})$ refers to the sum of the logarithms of the diagonal elements of the covariance matrix, given by $\sum_{d=1}^{k}\log\sigma_{d}^{2}\left(x_{i}\right)$.

### 4.2. GAN

**Figure 3:**
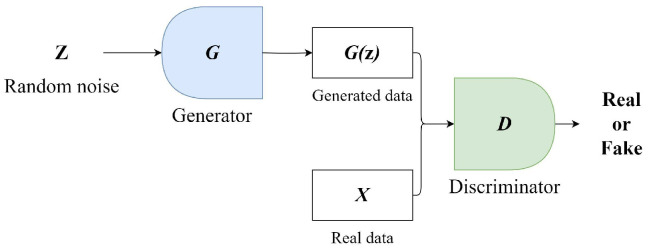
GAN architecture.

The GAN model was designed by Ian J. Goodfellow in 2014 [27]. GANs consist of two independently operating networks: a generator (*G*) and a discriminator (*D*). Figure 3 presents a simplified diagram of a GAN. The goal of the generator network is to learn and mimic the distribution of real data. It begins with a random noise vector *z* and tries to create samples that resemble real data. The discriminator is a binary classifier tasked with evaluating the output of the generator and differentiating between the fake data produced by the generator and actual real data. During training, the two networks are trained in parallel. The generator strives to produce data that appear more realistic over time, while the discriminator focuses on improving its ability to distinguish between real and fake data. This process involves a minimax game, where the discriminator tries to maximize its accuracy, while the generator aims to minimize the discriminator’s success. The ultimate aim is that through this competitive and learning process, the generator becomes adept at creating high-quality data indistinguishable from real data, while the discriminator becomes proficient at accurately identifying what is real and what is not [27].

Traditional GANs, while promising in generative modeling, often face issues with training instability, mode collapse, and vanishing gradients. Arjovsky et al. introduced WGAN, which effectively addresses these issues by adopting the Wasserstein distance as the objective function [28]. WGAN boasts superior theoretical properties, such as continuity and differentiability, leading to a more stable training process. The Wasserstein distance intuitively represents the minimum “cost” of transferring “mass” from one distribution to another, relying less on gradients, thus mitigating the vanishing gradient problem. This method provides meaningful results even when two distributions do not overlap on low-dimensional manifolds. The key to WGAN is maintaining the K-Lipschitz continuity of the discriminator during training, proposing a method of weight clipping after each gradient update to preserve this continuity. The Wasserstein GAN loss function is obtained by the Kantorovich–Rubinstein duality:

(3) $$\min_{G}\max_{D\in F}E_{x∼P_{r}}\left[D\left(x\right)\right]-E_{z∼p(z)}\left[D\left(G\left(z\right)\right)\right].$$

In this formulation, the generator *G* aims to minimize the estimated Wasserstein distance between the model distribution and the real data distribution, while the discriminator D∈F(a 1-Lipschitz function) seeks to maximize this distance. The term $E_{x∼P_{r}}\left[D\left(x\right)\right]$ denotes the expected discriminator score over real data samples, while $E_{z∼p(z)}\left[D\left(G\left(z\right)\right)\right]$ is the expected discriminator score over generated samples.

### 4.3. Diffusion Model

**Figure 4:**
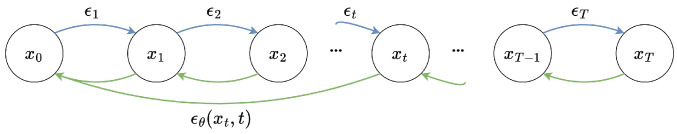
Denoised diffusion probabilistic model. Blue arrows represent the forward diffusion process, green arrows represent the reverse denoising process.

Diffusion models are also a type of latent variable model used for data generation, where the latent variables **$x_{1}$** to **$x_{T}$** have the same dimensions as the data **$x_{0}$** and represent hidden states that gradually change during the diffusion process. Diffusion models consist of two processes: the forward process and the reverse process. Figure 4 illustrates a simplified schematic of a denoised diffusion probabilistic model.

The forward process in the diffusion model is a fixed Markov chain that progressively adds Gaussian noise to the data, following a changing variance schedule $\beta_{1}$ to $\beta_{T}$. This process defines the transformation path of the data from its original state to a state of complete random noise. The distinction of diffusion models from other latent variable models lies in their use of an approximate posterior probability $q\left(x_{1:T}|x_{0}\right)$, which describes the process of reverting from completely noisy data back to the original data state:

(4) $$\begin{array}{cc} q\left(x_{1:T}|x_{0}\right) & =\prod_{t=1}^{T}q\left(x_{t}|x_{t-1}\right),q\left(x_{t}|x_{t-1}\right)\\ & =N\left(x_{t};\sqrt{1-\beta_{t}}x_{t-1},\beta_{t}I\right). \end{array}$$

In this equation, $q\left(x_{1:T}|x_{0}\right)$ represents the joint distribution of latent variables across all time steps, given the original data point $x_{0}$. This formula describes how we would gradually add noise to a noise-free data point. $q\left(x_{t}|x_{t-1}\right)$ is the specific conditional distribution, indicating that each step $x_{t}$ follows a normal distribution with $x_{t-1}$ as the mean and $\beta_{t}$ as the variance.

The reverse process of the diffusion model is a Markov chain starting from $x_{T}$, where each step is controlled by learned Gaussian transition probabilities. These transitions are modeled using Gaussian distributions, with $\mu_{\theta }$ and $\Sigma_{\theta }$ being parameters learned by the model. The full reverse process can be written as follows:

(5) $$\begin{array}{c} p_{\theta }\left(x_{0:T}\right)=p\left(x_{T}\right)\prod_{t=1}^{T}p_{\theta }\left(x_{t-1}|x_{t}\right)\\ p_{\theta }\left(x_{t-1}|x_{t}\right)=N\left(x_{t-1};\mu_{\theta }\left(x_{t},t\right),\Sigma_{\theta }\left(x_{t},t\right)\right)p_{\theta }\left(x_{0:T}\right). \end{array}$$

The reverse process starts from a predefined prior $p\left(x_{T}\right)=N\left(0,I\right)$. $p_{\theta }\left(x_{0:T}\right)$ denotes the joint distribution of all latent variables from time point 0 to T, where $\mu_{\theta }\left(x_{t},t\right)$,$\Sigma_{\theta }\left(x_{t},t\right)$ represent the specific conditional probability distributions modeled as Gaussian distributions parameterized by θ. $\mu_{\theta }\left(x_{t},t\right)$ is the conditional mean of the latent variable $x_{t-1}$ at time point t−1 given the latent variable $x_{t}$ at time point *t*, and $\Sigma_{\theta }\left(x_{t},t\right)$ is the conditional covariance matrix. These parameters are learned through the model to determine the transformation at each step of the reverse process.

A neural network, named the denoising U-Net, is trained to carry out the reverse diffusion process, predicting and removing noise from the noisy input to recover the original variables. The loss function for this process is expressed as follows:

(6) $$L_{DM}=E_{x_{0},\epsilon ∼N\left(0,1\right),t}\left[{∥\epsilon -\epsilon_{\theta }\left(x_{t},t\right)∥}^{2}\right],$$

where ϵ represents the true Gaussian noise, $\epsilon_{\theta }\left(\cdot \right)$ is the neural network that predicts the noise, and t∈{1,2,…,T} denotes the time step.

### 4.4. Classical Paradigms in BCI

In EEG-based BCI systems, classical paradigms such as motor imagery (MI), P300, and steady-state visual evoked potentials (SSVEPs) define the structure and characteristics of the neural signals to be processed. Although these paradigms were originally designed to facilitate specific communication or control tasks, they now also serve as practical categorizations for assessing EEG data generation techniques. Given the diversity of signal characteristics and task requirements across paradigms, generative models are typically evaluated within the context of one or more of these established BCI paradigms. In this section, we briefly introduce the most commonly used paradigms, which serve as the foundation for organizing the subsequent discussion on generative model applications.

Motor imagery (MI), as the name implies, is the process of mentally simulating an action in the brain without physically executing the action. According to studies by Jeannerod [29], MI can be considered as a conscious access to the content of motor intentions, which is typically carried out unconsciously during the preparation phase of movement. Jeannerod concluded that conscious motor imagery and unconscious motor preparation operate through the same mechanisms and are functionally identical. This explains why psychological practices using MI training can improve motor performance [29]. Due to the functional similarity between MI and motor execution (ME), the brain areas activated by both are highly overlapping. By analyzing EEG data and identifying the activation patterns in different brain regions, it is possible to determine the user’s intent, thereby achieving direct communication and control between the brain and external systems. Commonly focused areas for motor imagery include the left and right hands, both feet, and the tongue. During motor imagery, the cerebral cortex generates two types of rhythm signals with significant changes: the 8–15 Hz μ rhythm and the 18–24 Hz β rhythm. When engaging in motor imagery, neuron cells are activated, metabolism speeds up, and the electrical rhythmic energy in the contralateral motor sensory area of the cerebral cortex is significantly reduced, whereas that of the ipsilateral motor sensory areas is increased. This phenomenon is known as event-related desynchronization (ERD) and event-related synchronization (ERS). Therefore, a variety of control commands can be generated by actively controlling the amplitude of the μ and β rhythms in the left and right brain.

Emotion is a complex psychological state manifested through physical behavior and physiological activities [30]. When an organism perceives a situation that necessitates a response, automatic psychological and physiological reactions occur. Emotions impact both individuals’ health and their decision-making processes. There is currently extensive research being conducted in the field of emotion recognition, which is a crucial branch of affective computing and plays a significant role in understanding people’s thoughts and behaviors. Based on the type of signals used, emotion recognition can be categorized into two types: non-physiological signals and physiological signals. Non-physiological signals include vocal tone, body posture, gestures, facial expressions, and other similar signals. Physiological signals include electroencephalography (EEG), body temperature (T), electrocardiogram (ECG), electromyography (EMG), galvanic skin response (GSR), and respiration (RSP). Emotion recognition technology aims to identify two main parameters behind emotions: valence, which represents the change from unpleasant to pleasant, and arousal, which measures the change from calm to excited [31].

Brain activity is often influenced by external stimuli, such as flashing LEDs and sounds. The altered EEG activity can be collected and decoded to control real or virtual objects or external prosthetics [32]. The most commonly used external stimulation paradigms are the P300 and steady-state visual evoked potential (SSVEP) paradigms. This is because they both exhibit high signal response and signal-to-noise ratio. The classification accuracy rate and signal detection time impact the overall performance of BCI systems. These metrics are crucial for calculating the information transfer rate (ITR), a key performance indicator for a BCI system. A BCI system based on P300 or SSVEP typically has higher ITR values than other types of BCI systems [33].

The P300 is among the most widely studied event-related potentials (ERPs). It can be detected by averaging EEG signals in response to specific events. The reaction to unusual stimuli triggers the P300 component, a positive peak in ERP, typically ranging from 5 to 10 microvolts, occurring 220 to 500 milliseconds after the event. P300 recognition has been instrumental in developing essential communication tools and devices for patients with motor neuron diseases. BCI systems utilizing P300 provide these patients with affordable, portable, and non-invasive communication devices, potentially enhancing their quality of life. Despite advances in P300-based BCI, challenges remain in detecting and interpreting P300 signals. These waveforms are often high-dimensional and have a low signal-to-noise ratio (SNR). Additionally, P300 signals are known to be non-stationary, exhibiting significant variability across subjects [33].

SSVEP offers advantages such as minimal training requirements, high classification accuracy, and a high information transfer rate (ITR), making it widely regarded as one of the most effective paradigms for high-throughput BCI. SSVEP stands for steady-state visual evoked potential, which is an oscillatory response generated in the brain when a person views a visual stimulus with a frequency of 6 Hz or more. The brain’s natural oscillations may respond to such a stimulus. These SSVEP signals are most noticeable in the occipital region (visual cortex), with their primary frequency corresponding to the stimulus and its harmonics [34].

Epilepsy is a disease caused by abnormal excitation of brain cells leading to unprovoked seizures, with some main causes being hypoglycemia, malformations, and hypoxia during birth [35]. Epileptic seizures can occur at any time, leading to loss of consciousness and potentially resulting in injury or even death. Generally, epileptic seizures are classified into two main types: generalized seizures and partial seizures, depending on whether the seizure affects a part or all of the brain areas. In generalized seizures, all parts of the brain are affected; in partial seizures, only a specific region of the brain is involved [36]. Currently, for many patients with epilepsy, pharmacological treatments are not always effective, making it crucial to predict the occurrence of these seizures.

In addition to the widely adopted paradigms such as motor imagery (MI), P300, and SSVEP, several other BCI paradigms have been proposed to expand the scope and flexibility of EEG-based systems. These include paradigms based on real movement, somatosensory stimulation, and auditory and olfactory stimuli, as well as covert and overt attention. Other less common but potentially valuable approaches include observation-based paradigms, slow cortical potentials (SCPs), reflexive semantic conditioning, and passive paradigms where brain activity is recorded without any explicit task [32]. While these paradigms have received comparatively less attention in the context of EEG data generation, they offer promising avenues for future exploration, particularly in hybrid systems or personalized BCI applications.

### 4.5. Evaluation Metrics

To comprehensively assess the performance of generative models applied to EEG data, various evaluation metrics have been used in the literature. These metrics can be broadly categorized into four groups based on their underlying purpose and analytical focus: (1) Downstream task performance, which refers to how well the generated data support specific EEG analysis tasks such as classification, regression, or brain–computer interface (BCI) applications. High performance in these tasks indicates that the synthetic data preserve essential discriminative features of the original EEG. (2) Generative quality, focusing on how realistic and diverse the generated EEG signals are. Common metrics in this category include Fréchet Inception Distance (FID), Inception Score (IS), and signal quality indices that quantify the fidelity and variability of generated samples. (3) Task-specific metrics, which evaluate how well the generated data fulfill the specific requirements of particular EEG applications. For example, in brain–computer interface (BCI) systems, information transfer rate (ITR) is commonly used to measure the efficiency of information transmission. (4) Interpretability and visualization, which involve qualitative assessments such as visual inspection of waveforms, topographic maps, or latent space projections (e.g., t-SNE), helping to understand what the generative model learns and how it represents EEG data.

This categorization facilitates a more nuanced understanding of each study’s contributions and evaluation focus. Table 2 and Table 3 summarize the evaluation metrics used in this review.

## 5. VAE for EEG

Variational Autoencoders (VAEs) and Generative Adversarial Networks (GANs) are considered among the most valuable methods in the field of unsupervised learning, with more and more applications in the domain of deep learning. In this section, we first introduce the basic theoretical knowledge about VAEs, followed by a systematic review and summary of the recent applications of VAEs in the field of EEG generation.

### 5.1. Review of Related Work

In this section, we reviewed the various applications of VAEs in EEG generation, and Table 4 summarizes all the papers on the use of VAEs across various EEG tasks.

#### 5.1.1. VAE Models for Motor Imagery

Ozan et al. demonstrated the feasibility of using Conditional Variational Autoencoders (cVAEs) to generate multi-channel EEG signals under specific conditions [43]. The cVAE includes a stochastic encoder and a deterministic decoder. The encoder learns the variational posterior distribution of the latent representation, reconstructs the input signal through the decoder, and generates new samples during inference using a conditional variable. The data used in the experiments came from the PhysioBank EEG Motor Imagery Dataset, considering data from 100 participants who performed motor imagery tasks involving the right hand, left hand, and both feet. The experimental results indicate that the generated EEG segments can exhibit spectro-temporal characteristics under specific task conditions, including event-related desynchronization (ERD) patterns in the α and β bands for different tasks such as right-hand, left-hand, and both-feet movement imagery. These generated signals show significant frequency and spatial distribution differences across different task conditions.

Yang et al. proposed a model that combines Conditional Variational Autoencoders (CVAEs) and GAN for the generation and recognition of four classes of motor imagery EEG signals [44]. The CVAE-GAN integrates the encoder–decoder network of CVAE with the generative model of GAN. The encoder learns the latent representation *z* of data *x* under the condition of a specific class *y*, and the decoder uses this to predict the class. The GAN’s generator and discriminator work to produce data that approximate the real data distribution and distinguish between real and synthetic data. This study utilized both private [44] and public (BCI competition IV 2a) datasets. The authors processed the data by short-time Fourier transform (STFT) to convert the MI-EEG signals into a series of time–frequency images. The authors compared the model’s performance with other models (CVAE, CNN, CGAN) using metrics such as Inception Score (IS), Fréchet Inception Distance (FID), and Sliced Wasserstein Distance (SWD). The experimental findings indicate that CVAE-GAN performed best in terms of IS and SWD metrics, while CNN performed better in the FID metric but did not perform well in the other metrics. The augmented training dataset significantly improved the MI-EEG recognition performance, with experimental results showing that CVAE-GAN achieved higher classification accuracy across different datasets. In addition, the authors found that the generated data also exhibited significant power decreases and increases in the same electrode regions as the real data, corresponding to ERD/ERS phenomena.

George et al. detailed the study of data augmentation strategies in EEG-based decoding of motor imagery, where six data augmentation methods were employed to synthesize EEG motor imagery experimental data with the goal of improving decoding performance [47]. These methods include Trial Averaging (AVG), Temporal Slicing (RT), Frequency Slicing (RF), Noise Addition (NS), Cropping (CPS), and Variational Autoencoder (VAE). The generated data were assessed using four metrics: predictive accuracy, FID, t-Distributed Stochastic Neighbor Embedding (t-SNE) plots, and topographic head maps. The study showed that synthetic data shared similar characteristics with real data, and applying these methods resulted in an average accuracy increase of 3% and 12% on two public datasets, respectively. After applying augmentation techniques to the two datasets, the accuracy improvement with VAE compared to other augmentation methods was not significant. Additionally, VAE showed higher FID values compared to some other methods (e.g., adding noise), indicating that the data generated by VAE were slightly less similar to the statistical distribution of the original data. Furthermore, VAE was the most computationally expensive among all augmentation techniques, suggesting a need for a trade-off consideration when using VAE as an augmentation method in resource-constrained situations.

Zancanaro et al. introduced a model called vEEGNet, which is not only used for classification tasks in motor imagery but also for generating EEG signals [53]. vEEGNet comprises VAE and a feed-forward neural network. It utilizes the EEGNet [56] architecture within the VAE, with the encoder comprising temporal convolution, spatial convolution, and separable convolution, followed by a fully connected layer. vEEGNet’s decoder mirrors the encoder structure, employing transposed convolutions and upsampling layers. The feed-forward neural network is employed to classify EEG signals, with the output layer containing four neurons, each corresponding to one of the four motor imagery task categories. The model was tested on the BCI Competition IV 2a dataset, achieving an average classification accuracy of 71.95% and a kappa score of 0.63 across all subjects. The performance of vEEGNet was comparable to that of the other models, with accuracies ranging from 70% to 80% and a standard deviation of 8.78%, suggesting that performance varied considerably between subjects. Additionally, the reconstructed signals could identify motor-related cortical potentials (MRCPs), which are specific EEG components closely associated with motor execution or imagery.

#### 5.1.2. VAE Models for Emotion Recognition

Luo et al. introduced sVAE to solve the problem of data scarcity in emotion recognition [39]. This model adds a data augmentation strategy to the standard VAE. Two strategies were compared: one involving the complete use of all generated data, and the other focusing solely on the highest-quality synthetic data. To evaluate the quality of the data, classifiers trained on the original dataset (Support Vector Machine (SVM) or Deep Neural Network (DNN) with shortcut layers) were used to classify the generated data. The experimental results demonstrated that using sVAE with a DNN classifier, the accuracy rates on SEED and DEAP datasets increased by 4.2% and 4.4%, respectively, compared to the baseline.

Bao et al. introduced a data augmentation model called VAE-D2GAN [45]. This model extracts emotional features from EEG signals using Differential Entropy (DE) and transforms these features into topological images as inputs. It includes an encoder, a generator, and dual discriminators ($D_{1}$ and $D_{2}$). The encoder maps real samples into the latent space, the generator creates artificial samples based on the latent vectors, and the discriminators $D_{1}$ and $D_{2}$ distinguish between real and generated samples. The model’s performance was evaluated on two public emotional EEG datasets, SEED [57] and SEED-IV [58]. After using data enhancement, the recognition accuracy reaches 92.5% for SEED and 82.3% for SEED-IV, which are 1.5% and 3.5% higher than the recognition accuracy without data enhancement, respectively.

Bethge et al. introduced a model named EEG2Vec, which utilizes a framework based on Conditional Variational Autoencoders (cVAEs) aimed at learning generative–discriminative representations from EEG [46]. This method can predict emotional states and generate synthetic EEG data related to specific participants or emotions. EEG2Vec has three main components: a feature encoder, a decoder, and a classifier. The authors used EEGNet as the backbone network of the feature encoder. The latent representations obtained after data processing by the encoder are reconstructed in the decoder, and the classifier is used to predict emotional states. The final experimental results demonstrated that merging the original data with 20% synthetic data could increase the classification accuracy from 66% to 69%. Moreover, by adding 20% synthetic EEG data to the training set, the model significantly enhanced emotion recognition accuracy in some participants. Specifically, for participants 12 and 11, the emotion recognition accuracy increased by 42.85% and 31.57%, respectively.

Wang et al. introduced a Multimodal Domain Adaptive Variational Autoencoder (MMDA-VAE) approach, aimed at addressing the issue of limited calibration samples in EEG-based emotion recognition [50]. MMDA-VAE constructs a Multimodal Variational Autoencoder (MVAE) that projects multimodal data into a shared space. Through adversarial learning and cycle consistency regularization, it reduces the distribution differences across domains within the shared latent representation layer, thereby facilitating knowledge transfer. On this shared latent space, MMDA-VAE further trains a cross-domain classifier for emotion state recognition. The experiments were carried out using two datasets, SEED and SEED-IV. The results showed that MMDA-VAE significantly outperforms other modality fusion methods, such as Feature Level Fusion (FLF) based on SVM and Bimodal Deep Autoencoder (BDAE), achieving an average accuracy of 89.64% on the SEED dataset and 73.82% on the SEED-IV dataset. MMDA-VAE shows higher average accuracy on both datasets compared to common domain adaptation methods (e.g., TCA, DDC, DAN, etc.). The authors also conducted experiments on cross-subject emotion recognition, demonstrating MMDA-VAE’s good performance in handling cross-subject domain adaptation problems, with an average accuracy of 85.07% on the SEED dataset and 75.52% on the SEED-IV dataset.

Ahmed et al. utilized a deep learning model known as CNN-VAE to interpret and visualize the disentangled representation of individual-specific EEG topographic maps [54]. The main contribution of this study is to interpret the disentangled representation of VAE by activating only one potential component while setting the rest of the components to zero (since zero is the mean of the distribution), thereby identifying the role of each component in capturing the generative factors within the topographic maps. The experiment used the DEAP dataset, which contains multi-channel EEG recordings of 32 participants while watching 40 one-minute music video clips [59]. The researchers transformed the original EEG signals into EEG topographic maps that retained spatial information, further generating 40 × 40 sized EEG topographic head maps. Experimental results showed that when using all latent components as input for the decoder, metrics such as Mean Absolute Error (MAE), Mean Squared Error (MSE), Structural Similarity Index (SSIM), and Mean Absolute Percentage Error (MAPE) approached ideal values, indicating good performance of CNN-VAE in EEG topographic image reconstruction. In addition, the researchers found that by training the decoder by activating only one latent variable component and setting the others to zero, each latent variable made a different contribution to capturing the generative aspects in the topographic maps.

Tian et al. introduced a model named DEVAE-GAN, which integrates spatiotemporal features to generate high-quality artificial EEG data, aiming to address the current problem of data scarcity in EEG-based emotion recognition tasks [55]. DEVAE-GAN combines VAE and GAN, with a special emphasis on introducing dual encoders to concurrently capture the spatiotemporal characteristics of EEG signals. One encoder is designated for extracting processed time features, while another encoder is tasked with extracting spatial information distributed according to EEG electrode locations. These two types of information are considered latent variables. By merging these two vectors, a new latent variable distribution is formed, which contains complementary information. The authors conducted experiments using the SEED dataset, which contains EEG signals from fifteen subjects, categorized into positive, negative, or neutral emotions. The experimental results show that the method achieved an average classification accuracy of 97.21% on the SEED dataset, which is a 5% improvement over the original dataset. Additionally, the authors measured the similarity between the generated data and the original data using various metrics such as JS (Jensen–Shannon) divergence, KL divergence, and WD (Wasserstein distance). The results indicate that DEVAE-GAN performs the best in generating high-quality artificial samples that are similar to the distribution of the original data.

#### 5.1.3. VAE Models for External Stimulation

Aznan et al. utilized VAE as generative model to generate EEG signals, aiming to enhance the performance of steady-state visual evoked potential (SSVEP) classification [37]. The authors trained each generative model solely on the Video-Stimuli dataset under a single experimental condition. The VAE used 1D convolutions to encode features from given EEG data samples, which were used to parameterize a Gaussian distribution. Latent representations sampled from this distribution were passed to the decoder part of the model, which transformed the latent representations back into the original EEG data. The authors assessed the feasibility of improving classification results by using synthetic data, merging synthetic data (30 samples per class) with real data to form a training set. The results indicated that including synthetic data positively influenced the generalization ability across different subjects. In addition, the study revealed that pre-training on synthetic data improved the cross-subject generalization of the model, especially when pre-training with data generated by VAE, where there was a significant improvement in classification accuracy.

#### 5.1.4. VAE Models for Epilepsy

Li et al. proposed a model named CR-VAE for generating medical time series data [51]. Unlike traditional recursive VAEs, this model uses a multi-head decoder, where each decoder head is in charge of producing a different dimension of the data. By applying a penalty that induces sparsity to the weights of the decoder and encouraging certain weights to be zero, this model learns a sparse adjacency matrix that encodes causal relationships between all pairs of variables. This matrix allows the decoder to strictly follow the principles of Granger causality, making the data generation process transparent. The EEG dataset used by the authors is a real intracranial EEG recording dataset from patients with drug-resistant epilepsy. Compared with TGAN, VRNN, and VRAE, CR-VAE showed better performance both qualitatively and quantitatively.

#### 5.1.5. VAE Models for Other EEG Applications

Krishna et al. introduced an RNN-based VAE that enhances the performance of EEG-based speech recognition systems by generating more meaningful EEG features through a novel constrained loss function [40]. Unlike previous speech recognition methods, this model does not depend on extra features such as acoustic or phonetic features. Building upon the VAE framework, the model includes an additional component in the loss function (ASR model loss), enabling the generation of more significant EEG features directly from the raw data, thereby improving the performance of the speech recognition system. The model’s encoder employs a single-layer Long Short-Term Memory (LSTM) network to transform input EEG features into latent representations. The authors then connect the output of one dimension (the fifth dimension) to the ASR model to guide the VAE in generating EEG features most relevant to speech production. The authors conducted experiments on both isolated speech recognition and continuous speech recognition using datasets from [41,42]. For isolated speech recognition, the classifier model trained and tested with EEG features generated by the VAE model showed a performance improvement of 4.55% over the baseline model using original 30-dimensional EEG features. For continuous speech recognition, experiments using EEG features generated by the VAE model consistently outperformed the baseline, and the word error rate (WER) was lower than previous methods for the larger test set corpus size.

### 5.2. Conclusions

This section summarizes the VAE models used in EEG tasks, demonstrating remarkable effectiveness across a range of EEG applications. VAE provides a solid probabilistic framework by learning latent representations of raw data to generate new data samples. By compressing high-dimensional EEG data into low-dimensional latent representations, VAE reduces computational costs and speeds up calculations. It is particularly useful for understanding and modeling complex biological signals like EEG. Compared to traditional autoencoders, the key improvement of VAE lies in modeling latent variables as an isotropic Gaussian distribution. Due to the independence of its latent variables, VAE can better capture and generate the features of complex data like EEG.

Despite VAE’s good performance in generating EEG data, the quality of the generated data is still inferior to that of real data and data generated by GAN models. The generated data may contain some noise and inaccurate signal features, which can affect the performance of downstream applications. Additionally, VAE performs poorly when dealing with complex EEG data, such as multi-channel EEG data or high-frequency signals. Most importantly, although VAE models have shown excellent performance in various EEG tasks, the understanding of how they recognize and generate specific EEG patterns is still insufficient. Future research could focus on improving the interpretability of these models.

## 6. GAN for EEG

As one of the hottest deep learning models currently, GANs (Generative Adversarial Networks) are applied in various fields. This section systematically reviews the various applications of GAN models in the field of EEG generation.

### 6.1. Review of Related Work

In this section, we reviewed the various applications of GANs in EEG generation. As in Section 5, the reviewed studies are also categorized into five groups: motor imagery, emotion recognition, external stimulation, epilepsy detection, and other EEG applications.

#### 6.1.1. GAN Models for Motor Imagery

Currently, EEG-based motor imagery signals have been utilized in a range of healthcare applications, such as neurorehabilitation [29], restoring lost or damaged limb functions through the control of prosthetics or exoskeletons, replacing walking functions for those unable to walk with robotic wheelchairs, and for spelling and cursor control. However, MI-EEG signals are intricate and possess high-dimensional structures. Consequently, it is necessary to employ advanced machine learning and deep learning (DL) algorithms to analyze and interpret these brain data. Generative deep learning models are often used to enhance and improve training data. Since Goodfellow et al. proposed GANs, there have been numerous advancements in generating image, audio, and video data, highlighting their significant potential for various applications. However, the potential of GANs has not yet been explored in dealing with the challenge of limited dataset sizes in EEG tasks. We review recent studies that have demonstrated employing GANs for data augmentation can significantly enhance the performance of MI-based BCI systems. Table 5 summarizes the studies of GAN models in motor imagery tasks.

In 2018, Abdelfattah et al. proposed the Recurrent Generative Adversarial Network (RGAN) [60]. This model shares the same design philosophy with conventional GAN models, but it incorporates Recurrent Neural Networks (RNNs) in the generator part. EEG signals are continuous time series where subsequent samples may be related to previous ones, indicating the presence of temporal dependencies. Such dependencies mean that a sample in the signal could be influenced by the preceding sample. RNNs are particularly suited for handling this type of data because they can capture the time dynamics and long-term dependencies in sequence data. The generator consists of four hidden layers: the initial two layers are RNNs that capture the temporal dependencies within the signals, followed by two fully connected (FC) layers, each comprising 128 neurons. The input to the generator is a noise vector matching the dimensions of the real signal, and the output is a generated signal sample that is then used as input for the discriminator. The authors conducted two experiments. The first one compared the RGAN with the Autoencoder (AE) and Variational Autoencoder (VAE). They assessed each model by measuring the reconstruction error, using a 5-fold cross-validation to separate the dataset, and averaged the results across 109 subjects. The results indicated that the RGAN attained an average signal reconstruction accuracy of 89.8% ± 3.5% across the dataset of 109 subjects. In contrast, the average signal reconstruction accuracy for AE and VAE was 54.9% ± 6.5% and 69.9% ± 3.7%, respectively.

The second experiment (RGAN Augmentation under Reduced-Data Conditions) assessed how RGAN-based data augmentation affects the classification accuracy of three models: Deep Neural Network (DNN), Random Forest Trees (RFTs), and SVM [60]. For this assessment, the authors initially evaluated the mean and standard deviation of each model using 100% of the training data from each subject’s dataset. They then conducted two additional experiments with 25% and 50% of the original dataset size, respectively, while augmenting the remaining samples using the RGAN model. The performance of the different classification models (DNN, SVM, RFT) significantly improved with RGAN augmentation. With 25% of the dataset size and RGAN augmentation, the classification accuracy (mean ± standard deviation) for the three models was 82.3%±4.6%, 68.5%±5.2%, and 75.2%±6.1%. When using 50% of the dataset and augmenting with RGAN, the DNN’s performance was notably superior to that of SVM and RFT, with increases of approximately 21% and 14%, respectively. With RGAN-augmented datasets, even at smaller dataset sizes (25% and 50% of the original size), classification models were able to achieve performance close to that with 100% dataset size.

Hartmann et al. introduced a GAN named EEG-GAN, which is designed to generate EEG signals [62]. To address training stability issues, the researchers improved the training process of WGAN to make it more suitable for generating EEG signals. A key improvement in WGAN is the introduction of gradient penalty (GP), which resolves the issues caused by weight clipping in the original WGAN. Building on this, the authors further proposed a method to increase training stability by gradually relaxing the gradient constraints. Moreover, during the training process, they adopted a training approach that progressively increases resolution, starting from a lower resolution and gradually advancing to the target resolution. This method helps enhance the quality of the generated signals and reduces instability during training. They also employed advanced techniques such as batch normalization, equalized learning rate, and pixel normalization to further improve the model’s performance and stability. For EEG signal generation, the authors chose a conventional CNN. They explored different upsampling and downsampling methods, including nearest-neighbor upsampling, linear interpolation, and cubic interpolation, and compared their impacts on the quality of the generated signals. To assess the quality of the generated EEG signals, the authors used the following metrics: IS, FID, Euclidean Distance (ED), and SWD. The experiment utilized a dataset of EEG signals produced by subjects performing simple motor tasks (such as resting or moving the hand). These signals were recorded with a 128-electrode EEG system and downsampled to 250 Hz. Experimental results showed that EEG-GAN is capable of generating samples that closely resemble real EEG signals in both the time and frequency domains.

Zhang et al. proposed a Conditional Deep Convolutional Generative Adversarial Network (cDCGAN) to generate EEG signals for data augmentation [63]. The loss function of cDCGAN is similar to that of GAN but introduces conditional information during the generation and discrimination processes. During the generation process, the model first converts the EEG signals into time–frequency representations (TFRs) and uses a two-dimensional convolutional kernel to learn the time–frequency features. Then, it generates waveform EEG signals through the inverse process of wavelet transform. The authors evaluated the model using the BCI Competition II dataset III. Experimental results show that by adjusting the ratio of synthetic data to original data during mixed training, the classification accuracy improved from 85% (0.5) to 90% (2). Additionally, the authors compared the synthetic EEG TFR with the original EEG TFR; it can be seen that the synthetic data closely match the original data in major time–frequency features and includes some additional features.

Roy et al. proposed a novel approach, MIEEG-GAN, for generating motor imagery EEG signals in [64]. The model consists of two main components: a generator (*G*) and a discriminator (*D*), both constructed using Bidirectional Long Short-Term Memory (Bi-LSTM) neurons. The experiments utilized Dataset 2b from the BCI Competition IV [76]. The study compared the first-order features between generated and original EEG signals, finding similarities that indicate the GAN’s capability to capture the temporal relations inherent in real EEG signals. The authors conducted a short-time Fourier transform (STFT) analysis to compare the time–frequency characteristics of real and synthetic EEG data. The results showed that synthetic and real EEG signals exhibit similar power spectral density (PSD) characteristics in the β-band (13–32 Hz), although the synthetic signals were noisier than the real ones. Furthermore, the Welch method was used to analyze the PSD, revealing similar patterns of power decrease in specific frequency ranges between real and synthetic signals. This indicates that synthetic EEG signals can effectively simulate the statistical properties of real EEG signals. The authors noted that with more training trials involving motor imagery tasks, the model is expected to better learn key neural patterns such as event-related desynchronization (ERD) and event-related synchronization (ERS), thereby enhancing the utility of the synthetic signals for classification tasks.

Debie et al. proposed a privacy-preserving GAN for generating and classifying EEG data while protecting data privacy [65]. The main goal of this method is to generate realistic EEG signals without disclosing sensitive features of the original data. The experiments were conducted using the Graz dataset A, which includes data from nine healthy subjects performing four motor imagery tasks: imagining movements of the left hand, right hand, both feet, and the tongue [66]. Differential privacy, mentioned in the article, is a technique to protect individual privacy, especially during the publication and analysis of statistical databases. It protects personal information from being disclosed by ensuring that the output of an algorithm does not significantly change whether or not any individual’s data are included or removed from the database. In the process of synthesizing EEG data, the authors trained two models using Differential Privacy Stochastic Gradient Descent (DP-SGD): a non-private GAN (NP-GAN) and a privacy-preserving GAN (PP-GAN). NP-GAN is a GAN trained normally to generate synthetic EEG data without incorporating privacy measures. PP-GAN, on the other hand, integrates differential privacy techniques during the GAN training process to ensure that the generated data maintain high fidelity while protecting the privacy of subjects. Over 1000 training epochs, the loss functions of the generator and discriminator were monitored. The loss functions in the training of NP-GAN and PP-GAN indicated that the models gradually achieved the capability to generate high-quality synthetic data. The authors investigated the impact of the noise multiplier on the performance of PP-GAN, especially regarding the quality of generated data and privacy protection. A higher noise multiplier offers stronger privacy protection but may affect the quality of the generated data, making the appropriate selection of the noise multiplier key to balancing privacy protection and data quality. Increasing the training data to 150 artificially generated samples improved the classification accuracy of the utilized classifiers, while using 200 artificially generated samples resulted in poor outcomes.

Luo et al., in reconstructing EEG signals, did not employ the traditional time mean squared error but introduced a novel reconstruction algorithm based on GAN that incorporates Wasserstein distance [77] and a temporal–spatial-frequency (TSF-MSE) loss function [67]. The entire WGAN-EEG framework comprises three parts: a deep generator, a TSF-MSE loss calculator, and a discriminator network. In the first part, the authors employed the same layout of “B residual blocks” as proposed by He et al. [78], where 16 B residual blocks are applied to the original EEG signals to extract deep features of the generator. The second part is the TSF-MSE loss calculator, which inputs both the generator’s reconstructed EEG signals and the real signals to extract common spatial patterns (CSPs) and PSD features. Then, using the extracted features, the loss is calculated based on the TSF-MSE loss function, which decomposes the reconstruction error into three components: temporal MSE between time steps ($L_{T-MSE}$), which reflects signal continuity over time; spatial MSE between EEG channels ($L_{S-MSE}$), which measures spatial consistency across electrodes; and frequency MSE between signal batches ($L_{F-MSE}$), which captures the preservation of spectral information in the generated signals. The authors utilized three datasets: the Action Observation (AO) dataset [79], Grasp and Lift (GAL) dataset [80], and BCI competition IV dataset 2a [81]. Through experiments, the authors found that under the same sensitivity conditions, the similarity of reconstruction effects of the framework on different datasets is AO > GAL > MI, indicating that the high-sensitivity WGAN framework reconstructed low-sensitivity EEG signals well, but the low-sensitivity model could not accurately reconstruct high-sensitivity EEG signals. For reconstructions of the same sensitivity, the WGAN framework outperformed the GAN framework in terms of average spectral results and Brain Electrical Activity Mapping (BEAM) results. For reconstructions of different sensitivities, the high-sensitivity model performed better in terms of average spectral differences and BEAM. Specifically, the signals reconstructed by the WGAN framework exhibit more distinct ERD/ERS patterns in the BEAM results. According to quantitative analysis, the WGAN framework exhibited higher classification accuracy. The classification accuracy rates of the WGAN-reconstructed signals on the AO dataset, GAL dataset, and MI dataset were 67.67%, 73.89%, and 64.01%, respectively, showing improvements of 4.1%, 4.11%, and 2.03% compared to the original data, respectively.

Zhang et al. researched and compared different DA methods to improve the classification performance of MI data [21]. The study included traditional methods (geometric transformations, AE, VAE) as well as DCGANs. Compared to traditional GAN models, DCGANs replace the pooling layers with fractional-strided convolutions in the generator and strided convolutions in the discriminator. The authors utilized DCGANs to obtain spectral graphs of MI data, which were subsequently classified by CNN to verify the performance improvement after data augmentation. Two datasets were used: BCI competition IV datasets 1 and 2b, with evaluation metrics including FID, average classification accuracy, and average kappa value. Compared to the baseline, the average classification accuracy of the CNN method without data augmentation was 74.5% ± 4.0% for dataset 1 and 80.6% ± 3.2% for dataset 2b. Different DA methods (NA-CNN, VAE-CNN, and DCGAN-CNN) provided higher accuracy, with DCGAN-CNN’s classification accuracy being 12.6% higher than the baseline for dataset 2b and 8.7% higher for dataset 1. In dataset 1, the accuracy of the CNN-DCGAN model was 5% higher than the average accuracy of VAE and AE, thus outperforming the best classification method mentioned among the DA methods previously. Furthermore, in terms of classification accuracy for dataset 2b, DCGAN’s accuracy was 5.6% and 10% higher than VAE and AE, respectively.

In 2021, Fahimi et al. proposed a framework based on DCGAN for generating artificial EEG to augment the training set, thereby improving the performance of BCI classifiers [68]. During the training process, the authors first extracted feature vectors from a subset of the target participant’s data using a pre-trained Deep Convolutional Neural Network (DCNN) model. Then, they trained the GAN using these feature vectors as a condition to generate new EEG data. The generated EEG data were merged with the original training set to form an augmented training set for training the BCI system’s classifier. The experiment included 14 healthy participants aged between 21 and 29 years. Participants were asked to perform a motor task under two different conditions: a focused attention condition and a diverted attention condition, which included opening and closing the right hand. The authors used an end-to-end DCNN as the baseline classifier to classify the EEG data of participants under both focused and diverted attention conditions. The results showed that, without data augmentation, the classifier achieved an average accuracy of 80.09% under the focused attention condition, which dropped to 73.04% under the diverted attention condition. After augmenting with data generated by DCGANs, the classification performance significantly improved. Under the focused attention condition, the accuracy increased by 5.45% and it increased by 7.32% under the diverted attention condition. The study also compared the effects of data augmentation using Variational Autoencoders (VAEs) and time–frequency domain segmentation and recombination (S&R) methods. The results showed that the DCGAN significantly outperformed these two methods in the diverted attention condition.

Song et al. proposed a new framework based on GANs, named CS-GAN (Common Spatial GAN), aimed at improving the accuracy of cross-subject EEG signal classification by generating high-quality data [69]. Unlike traditional GANs’ discriminators, the discriminator of CS-GAN includes two modules: one focused on discriminating EEG signals, and the other focused on maintaining the spatial features of the EEG signals (referred to as the CS-M). This module utilizes a spatial filter to process the input EEG data and enhances the spatial properties of the data during the training process. The training of CS-GAN employs not only the traditional adversarial loss but also introduces two special loss functions: covariance loss (cov-loss) and eigenvalue loss (ev-loss), which are used to maintain the spatial patterns of the generated data similar to the original data and enhance the distinction between different categories, respectively. The experiments used the BCI competition IV dataset 2a, which contains EEG data of nine subjects performing four types of motor imagery tasks (left hand, right hand, both feet, and tongue). The experimental results show that using 100 real samples for adaptive training yields an average classification accuracy of 59.40%, while using 3000 generated samples achieves an average accuracy of 67.97%, which is 15.85% higher than the leave-one-subject-out (LOO) test result of 52.12%. Compared to other data augmentation methods, the classification accuracy of the CS-GAN method is also higher: adding Gaussian noise achieves 64.60%, segmentation and recombination achieves 64.54%, VAE achieves 55.73%, and DCGAN achieves 54.20%. The authors’ ablation experiments further validate the effectiveness of each module in the CS-GAN method. Removing all modules results in a classification accuracy of 56. 97%, removing the common spatial module (CS-M) results in 57.33%, removing the covariance loss (cov-loss) results in 65.54%, and removing the eigenvalue loss (ev-loss) results in 66.02%.

Xu et al. enhanced the dataset for classifying left and right hand motor imagery (MI) in stroke patients by generating additional electroencephalography (EEG) data using Cycle-Consistent Adversarial Networks (CycleGANs) [70]. The research team utilized the EEG2Image method based on the Modified S-transform (MST) to convert EEG data into EEG topographies, a method capable of preserving the frequency domain characteristics and spatial information of the EEG signals. MST improves upon the S-transform by introducing adaptive parameters to optimize the accuracy of high-frequency energy calculations, thereby better focusing on the time–frequency characteristics of EEG data. In data processing, researchers first downsampled the original EEG data, then used MST to extract activities in the mu and beta frequency bands, and converted this information into 2D images through polar projection. Subsequently, researchers employed CycleGAN to learn and generate stroke patients’ motor imagery EEG data. CycleGAN includes two generators (*G* and *F*) and two discriminators ($D_{x}$ and $D_{y}$), used for unpaired image-to-image translation between two data domains. In this study, one domain was the EEG topographies of healthy subjects, and the other was the EEG topographies of stroke patients. CycleGAN ensured consistency of information through cycle consistency loss when converting from one domain to another and then back again. Experiments compared the impact of adding different quantities of generated data on classification accuracy. When the amount of generated data equaled the original data volume, classification performance significantly improved. However, as more generated data were added, the increase in classification accuracy began to plateau, and in some cases, even slightly decreased. In addition, the authors found that the generated data also exhibited ERD characteristics in the μ rhythm.

Xie et al. proposed a novel algorithm that combines Long Short-Term Memory Generative Adversarial Networks (LGANs) and Multi-Output Convolutional Neural Networks (MoCNNs) for MI classification and introduced an attention network to enhance model performance [71]. The LGAN’s generator consists of a fully connected layer and four convolutional layers, aimed at generating realistic MI data and establishing a mapping between categories and data. The discriminator is composed of three convolutional layers, one LSTM layer, and a fully connected layer. MI data often exhibit strong temporal features, which are challenging to recognize with convolutional layers alone. The LSTM can extract temporal information from the MI data’s time series. Thus, the features extracted by the CNN are fed into the LSTM layer. The discriminator’s fully connected layer serves as the final output network. The MoCNN model includes a convolutional layer feature extraction network and three sub-classification networks. Specifically, the feature extraction network shares the same structure as the convolutional layers in the discriminator, and they share parameters. During the training of MoCNN, the feature extraction network is not trained, and the output of each convolutional layer is input into the sub-classification networks. Each sub-classification network completes the classification task based on the features it receives and then outputs the classification results. The authors used two datasets for experimentation: BCI competition IV dataset 2a and BCI Competition IV dataset 2b. The experimental results indicate that on dataset 2a, after data augmentation using the proposed model, the average classification accuracy reached 83. 99%, significantly higher than other generative adversarial network methods (e.g., InfoGAN at 75.30% and WGAN at 60.87%). On dataset 2b, the classification accuracy reached 94.31%, significantly outperforming other methods (e.g., FBCSP combined with CNN at 82.39% and attention network combined with CNN at 87.60%). Confusion matrix analysis showed that data augmentation increased the overall classification accuracy by approximately 8%, and the kappa value increased from 0.688 to 0.787. By evaluating the generated data using the correlation coefficient (R value) and mutual information score (I value), it was found that the data generated using this method had the highest similarity to the real data, especially in the left-hand and right-hand MI data.

Raoof et al. proposed a conditional input-based GAN model for generating spatiotemporal electroencephalograph (EEG) data related to motor imagery [72]. Besides the generator and discriminator, this model also includes an encoder and a decoder, which together form an autoencoder. This autoencoder is used to encode EEG data into a low-dimensional (i.e., latent) space and recover the original data from this space. All four components adopt Gated Recurrent Unit (GRU) structures to generate high-quality EEG data through conditional input. The authors conducted experiments using an EEG dataset comprising 10 subjects. Evaluation metrics include the inverted Kolmogorov–Smirnov test, KL divergence, classification experiments (LSTM, SVM, 1D TCNN), and visualization techniques (PCA, t-SNE). The experimental results show that the data generated by the model are highly similar to real data in terms of statistical properties, classification performance, and feature space distribution, achieving an accuracy of 94.1% in classification tasks. This significantly enhances the performance of motor imagery classification and also indirectly verifies that the synthetic data preserve frequency response features related to ERDs.

Dong et al. proposed an EEG data augmentation method based on DCGAN to address issues caused by data scarcity or imbalance in EEG data classification [73]. The DCGAN in this study uses one-dimensional (1D) convolutional layers to handle EEG time series data. The generator takes random Gaussian noise as input, which passes through two dense layers and Parametric Rectified Linear Unit (PReLU) activation functions, and then generates EEG data through five transposed convolution blocks with different kernel sizes and strides. The discriminator receives either real or generated EEG data as input, extracts features through five 1D convolutional blocks, each consisting of a 1D convolutional layer, batch normalization layer, and PReLU activation function. Additionally, a dropout layer is added after each convolutional block to prevent overfitting. Finally, two dense layers are used to determine the authenticity of the input data. The authors evaluated the model using the publicly available EEG Motor Movement/Imagery dataset from Physionet. The authors analyzed the generated data using Fast Fourier Transform (FFT) and Continuous Wavelet Transform (CWT) and found that the generated data were similar to the real data in terms of temporal and frequency characteristics. The frequency distribution trends of the generated data and the real data are similar, primarily concentrated in the range of 0–10 Hz. Additionally, the power spectral maps of the generated signals and the real signals match well within the range of 0–2.5 Hz and 0–1 s.

Yin et al. propose an unsupervised end-to-end subject adaptation method named GITGAN for EEG motor imagery analysis, aiming to improve cross-subject data adaptability in BCI systems through GAN, thereby enhancing the system’s generalization capability [74]. GITGAN combines strategies for outlier removal, data augmentation, and generative adversarial learning to facilitate EEG data transfer between different subjects. The entire process starts with data preprocessing, followed by a generator, encoder, and domain discriminator, and finally, classification in the target domain. In the outlier removal process, the authors propose cbaDBSCAN (class-balanced auto-adaptive DBSCAN). The improved DBSCAN algorithm clusters data separately for each class, dynamically adjusting clustering parameters such as the radius and the minimum number of neighbors (minPts) based on class characteristics to ensure reasonable clustering results. For data augmentation, the authors use the MixUp method to increase the data volume, mitigating the reduction in data caused by outlier removal. After data preprocessing, generative adversarial learning is employed. The encoder and generator structures transfer features from the source data to the target data domain, creating a target data-centered space that maintains the integrity of the target data’s latent representation. Additionally, a label consistency mechanism is introduced to ensure that the class information of the source data is preserved during transfer. Experiments conducted on the OpenBMI [75] and BCI Competition IV dataset 2a show that the accuracy, F1-score, and kappa value of GITGAN are significantly superior to those of other comparison methods (EEGITNet, EEGResNet, EEG-Adapt, ADAST, SLARDA, TSMNet). Through the Layer-Wise Relevance Propagation (LRP) technique, the authors also analyze the interpretability of the GITGAN model. LRP results show that GITGAN can accurately identify brain regions associated with motor imagery tasks.

#### 6.1.2. GAN Models for Emotion Recognition

Electroencephalography is a non-invasive physiological signal that directly measures brain activity under different emotional states [82]. Compared to other methods, EEG offers higher temporal resolution, faster data collection and transmission, and lower cost. Moreover, EEG is a spontaneous, objective physiological signal that can more accurately and intuitively reflect human emotional states. Despite these advantages, EEG also has limitations. One major challenge in using EEG for emotion recognition is the presence of various noises and artifacts, which can easily affect the EEG signals. Another drawback is its limited spatial resolution, which may hinder the accurate detection of specific brain regions activated under emotional states. Additionally, the process of obtaining EEG signals is often time-consuming, making emotion recognition from EEG a challenging and arduous task [82]. Traditional machine learning (ML) and deep learning (DL) techniques are the main methods to extend emotion recognition approaches based on EEG. Therefore, training an effective model to extract time, frequency, time–frequency, and non-linear features from preprocessed EEG signals is crucial. Due to the scarcity of EEG data, the performance of models can hit a bottleneck. In recent years, many researchers have used GAN models to expand the dataset and enhance model performance. Table 6 summarizes different studies that used GANs to achieve an improvement in the emotion recognition field.

Luo et al. utilized Conditional Wasserstein Generative Adversarial Networks (cWGANs) in their study for emotion recognition based on EEG signals [83]. CWGAN, a variant of GAN, is employed to generate more realistic data. In this research, it was used to create synthetic Differential Entropy (DE) features of EEG signals. These features mimic real EEG data, aiding in the improvement of emotion recognition model performance. Additionally, a gradient penalty term was added to stabilize the training process. The study employed three metrics to evaluate the quality of generated data: Wasserstein distance [77], Maximum Mean Discrepancy (MMD) [92], and two-dimensional mapping. Experimental results demonstrated that the emotion recognition framework trained with cWGAN-generated data and original data outperformed the framework using only original data by 2.97% on the SEED dataset and improved arousal and valence classification by 9.15% and 20.13%, respectively, on the DEAP dataset. Furthermore, the authors observed that the discriminator’s loss values quickly approached zero for each subject, indicating high quality of the generated data.

In their new study, Luo et al. proposed and compared two GAN-based models for EEG data augmentation: Conditional Wasserstein Generative Adversarial Network (cWGAN) and Selective Wasserstein Generative Adversarial Network (sWGAN) [39]. cWGAN generates data matching given conditions by incorporating emotion labels as conditional variables. sVAE is based on variational autoencoders for data generation, followed by a selection process to filter out the data most beneficial for improving the performance of the emotion recognition model. sWGAN, similar to sVAE, introduces a selection mechanism on top of WGAN. The authors utilized two datasets (SEED and DEAP), two types of features (PSD and DE), and various classifiers for their experiments. On the SEED dataset, without data augmentation, the average accuracy of the original training set was 60.3%. With the cWGAN method, when adding 15,000 samples, the average accuracy reached 65.2%. The sWGAN method performed the best, achieving an average accuracy of 67.7% when adding 20,000 samples. On the DEAP dataset, the average accuracy of the original training set was 42.7%. cWGAN achieved the best average accuracy of 45.0% after adding 5000 generated samples. The sVAE method reached the best average accuracy of 46.1% after adding 15,000 samples. Again, sWGAN was the top performer, achieving the highest average accuracy of 47.6% when adding 20,000 samples. Compared to traditional data augmentation methods, the approaches proposed in this study (especially sWGAN) were more effective in improving model performance.

Chang et al. proposed a hybrid deep learning model based on EEG aimed at recognizing users’ emotional reactions to architectural design proposals [84]. This model combines GAN for EEG data augmentation with an EEG-based deep learning classification model for data classification. The objective of the classification model is to categorize EEG data into “positive” or “negative” emotional reactions towards architectural design proposals. The classification model is implemented using TensorFlow and related application libraries, comprising three levels: input layer, hidden layers, and output layer. The study used the dataset from [85] of 18 subjects for evaluating the proposed model. Experimental results indicated that the data augmentation method led to an average increase of 10.92% in recognition accuracy for individual subject’s emotion recognition tasks and an average increase of 14.47% in recognition accuracy for cross-subject emotion recognition tasks.

Dong et al. proposed a multi-reservoir feature coding continuous label fusion semi-supervised learning model based on GANs, named MCLFS-GAN, for emotion recognition [86]. The MCLFS-GAN model uses a multi-reservoir encoder to perform convolutional optimization on the permutation phase transfer entropy features of EEG signals, generating feature expressions with time series relationships. By leveraging adversarial training between the generator and discriminator, the model generates pseudosamples similar to the distribution of real samples and achieves feature space invariance among different subjects through transfer learning. The authors employ a label fusion method to transform discrete emotion labels into continuous labels, enabling the model to learn varying degrees of emotional information and enhancing its stability. The authors tested the emotion recognition performance of different algorithms using the DEAP dataset, including CNN + LSTM, L1-norm + SVM [93], SAE + LSTM [94], GELM (Graph Extreme Learning Machine) [95], DANN (Domain-Adversarial Neural Network) [96], and ACGAN (Auxiliary Classifier GAN) [97]. Two implementation schemes are used to test the recognition performance of the model. The first scheme involves randomly shuffling all samples followed by a 10-fold cross-validation (SAP). The second scheme is the leave-one-subject-out (LOSO) method [93]. In the cases of SAP and LOSO, the best classification accuracies based on brain region partitioning were 81.32% and 54.87%, respectively. The authors compared the recognition rates under different algorithms, where MCLFS-GAN achieved a recognition rate of 81.32% under SAP and 54.87% under LOSO, showing a significantly better overall recognition effect compared to other models.

Zhang et al. employed a Multiple Generator Conditional Wasserstein GAN (MG-CWGAN) approach to enhance emotion recognition based on EEG data [87]. This method generates high-quality artificial data capable of covering a more comprehensive distribution of real data. The model consists of multiple generators and a single discriminator, with each generator responsible for producing data of a specific type or feature. These generators share most parameters to reduce computational load and share underlying information, but their input layers are independent to allow for the reception of different noise vectors and conditional labels. During the training phase, each generator receives a joint input composed of a noise vector and a conditional label, with the conditional label guiding the generator to produce EEG data of a specific type. To enhance the model’s stability and convergence speed, the authors modified the gradient penalty term (from a one-centered gradient penalty to a zero-centered gradient penalty), significantly enhancing the network’s convergence performance in practice. The authors conducted experiments using the SEED dataset and assessed the quality of the generated data through methods such as MMD, Wasserstein distance, t-SNE, and semi-supervised self-training. The results showed that the data generated by the MG-CWGAN are closer in distribution to real data, with the Wasserstein distance fluctuating within the range of [0.2, 0.4] after training stabilization, indicating a smaller distance between the generated and real data. The authors used SVM and K-Nearest Neighbors (KNN) as classifiers to assess the accuracy of the emotion recognition model after adding different quantities of generated data. The results demonstrated that with an increase in the amount of generated data, both SVM and KNN performance improved. Especially when adding up to 15,000 generated data points, the classification accuracy of SVM increased by approximately 1–2% from the baseline of the original training set.

Liang et al. developed a model named EEGFuseNet, a hybrid unsupervised deep learning approach for feature extraction and fusion, specifically designed to handle high-dimensional EEG data, particularly for applications in emotion recognition [88]. This model leverages the strengths of CNNs, RNNs, and GANs to autonomously extract spatial and temporal dynamic features from EEG signals. The model first uses multiple convolutional layers to extract spatial features from raw EEG signals, capturing activity patterns from different brain regions. It then captures the temporal dynamic features of the EEG signals using bidirectional GRUs. Finally, the extracted features are input into a GAN, where the generator and discriminator are adversarially trained to enhance the quality of feature representations. EEGFuseNet’s unsupervised training mechanism does not require labeled data and achieves cross-subject emotion classification through a hypergraph decoding model [98], effectively addressing individual differences. The authors evaluated the EEGFuseNet model on three commonly used EEG emotion databases: DEAP, MAHNOB-HCI, and SEED, using accuracy, F1-score, and Normalized Mutual Information (NMI) as evaluation metrics. The experimental results show that on the DEAP database, the accuracy for various emotion dimensions ranges from 56.44% to 65.89%, and the F1-score ranges from 70.83% to 78.46%. On the MAHNOB-HCI database, the accuracy ranges from 60.64% to 74.63%, and the F1-score ranges from 62.05% to 83.61%. On the SEED database, the accuracy for three-class emotion classification is 59.06%, and for two-class classification, the accuracy is about 80%. The results demonstrate that the proposed hybrid EEGFuseNet (CNN-RNN-GAN-based) outperforms the other networks (CNN-based, hybrid CNN-GAN, and hybrid CNN-RNN).

Pan et al. introduced a model named PSD-GAN, which utilizes GANs and CNNs to address the issues of insufficient samples and sample class imbalance [89]. PSD-GAN is designed to generate samples with PSD features. The network’s input is a 1×16-dimensional random noise, which the generator uses to produce a fake sample. The generator is composed of three linear layers and three activation functions (two ReLU functions and one Tanh function). The discriminator’s structure comprises two linear layers and two activation functions, including one LeakyReLU function and one Sigmoid function. The researchers used the DEAP dataset to validate the performance of PSD-GAN. Experimental results showed a significant improvement in model recognition accuracy after data augmentation. Particularly, in the four-class task, the data augmentation method increased the recognition accuracy of individual subjects by an average of 10.92% and cross-subject recognition accuracy by an average of 14.47%. The study also designed two comparison models: Frequency Band Correlation Convolutional Neural Network (FBCCNN) and Frequency Band Separation Convolutional Neural Network (FBSCNN), to explore how the correlation features between frequency bands affect emotion recognition. The experimental results show that after data augmentation using PSD-GAN, the emotion recognition accuracy of both FBCCNN and FBSCNN significantly improved in the two-class and four-class tasks.

Liu et al. proposed a task-driven method based on Conditional Wasserstein Generative Adversarial Networks (CWGANs) to generate high-quality artificial EEG data for improving the performance of EEG-based emotion recognition tasks [90]. Building on the GAN model, CWGAN introduces conditional information to generate data under specific conditions, thus achieving more precise data generation. The experiment utilized the DEAP dataset. The original EEG signals were first decomposed into four frequency bands (θ, α, β, and γ), from which Differential Entropy (DE) features were extracted. These features were then mapped into a two-dimensional feature matrix and constructed into a 4 × 9 × 9 feature cube based on the spatial distribution of EEG channels. The experimental results of this study were assessed in two main aspects: the quality of the generated data and the improvement in the performance of the emotion recognition task. The results showed that, compared to the original CWGAN, the method proposed in this paper achieved lower W-distance and MMD values in two emotional dimensions (arousal and valence), indicating that the generated data are closer to the real data in distribution. Moreover, SVM and three different neural networks were selected as classifiers to evaluate the accuracy of the emotion recognition task using original data, data augmented with the original CWGAN, and data augmented with the proposed method. The results revealed that, compared to the non-augmented data and the data augmented with the original CWGAN, the models trained with data augmented by the proposed method showed significant improvement in accuracy in both emotional dimensions (arousal and valence), with an increase in accuracy ranging from 1.5% to 5.5%.

Qiao et al. designed an EEG emotion recognition model based on attention mechanisms and GANs [91]. This study aims to enhance raw EEG data through the superposition of spatial attention and channel attention, thereby addressing the issues of weak features and susceptibility to interference in EEG data. Initially, the authors constructed an emotion cognitive map of the brain by extracting graphical features from EEG signals. Then, using the GAN model to generate additional data, the authors combined spatial and channel attention to process the data, improving the expressiveness of the features. Finally, the data enhanced by the attention mechanism were input into a Convolutional Recurrent Neural Network (CRNN) for emotion classification. Among different machine learning algorithms, the study compared the performance of CNN, SVM, LSTM, and CRNN on processing EEG data enhanced by the attention mechanism. The CRNN algorithm combines the image feature extraction capabilities of CNN and the sequential data processing advantages of RNN, resulting in optimal performance with a recognition accuracy of 73% and a reduced computation time of 2.36 min. Experiments were conducted on two commonly used emotion datasets, SEED and DREAMER, with the model achieving a recognition accuracy of 94.87% on the SEED dataset [58] and 87.26% on the DREAMER dataset [99].

#### 6.1.3. GAN Models for External Stimulation

In recent years, numerous studies have demonstrated the potential of using GANs to address the issue of data scarcity in tasks based on the external stimulation paradigms. Table 7 summarizes the studies of GAN models in external stimulation.

Panwar et al. proposed a method based on Conditional Wasserstein Generative Adversarial Network with Gradient Penalty (cWGAN-GP) for synthesizing EEG data to support data augmentation for different cognitive events in Rapid Serial Visual Presentation (RSVP) experiments [100]. The model consists of a generator and a discriminator. In the generator, a 120-dimensional sample drawn from a normal distribution is reshaped into a structure with 128 channels after passing through two fully connected layers, followed by processing through bi-cubical interpolation upsampling, batch normalization, and LeakyReLU activation function. This is then followed by convolutional and deconvolutional layers, where the authors used bilinear weight initialization in the deconvolutional layers to avoid artifacts in the generation process.

The experiments first trained the Wasserstein GAN (WGAN) model on target and non-target samples separately to construct a robust GAN model architecture for specific EEG data. The experiments utilized bi-cubical interpolation (BC) and deconvolution (DC) with bilinear weight initialization to generate signals and address the issue of artifacts. By comparing the log-likelihood values of samples generated by different GAN architectures with real samples, the BCDC architecture showed log-likelihood values closer to those of real samples for both target and non-target samples. The authors trained the cWGAN-GP model with embedded class labels to avoid mode collapse. The results indicated that the generated target samples showed visible ERPs (event-related potentials) at about 300 ms, while the generated non-target samples, although not showing visible ERPs, successfully captured the 5 Hz image presentation rate. Finally, the authors combined the generated samples with real samples for data augmentation. The performance of the augmented data and the quality of the generated samples were evaluated by training two CNN classifiers (one with real training data and the other with augmented data). The results showed that the classifier trained with augmented data performed better on real test data samples than the classifier trained only with real data, both in within-subject and cross-subject evaluations.

In another study by the research team, a novel Wasserstein Generative Adversarial Network (WGAN-GP) architecture was proposed to generate high-quality multi-channel EEG data [101]. This architecture was further extended to a Class-Conditioned WGAN-GP (CC-WGAN-GP) to optimize EEG event classification performance. The WGAN-GP framework consists of a generator and a discriminator, and it improves training stability and convergence by calculating the Wasserstein distance between real data distribution and generated data distribution, and introducing gradient penalty. The CC-WGAN-GP framework incorporates class labels as conditions and includes an event-related classification branch, enabling it to not only generate data but also optimize classification performance. To address challenges in generating EEG data, such as frequency artifacts, the authors adopted a two-step upsampling method that combines bicubic interpolation with deconvolution and bilinear weight initialization, effectively enhancing the quality of the generated samples. The study utilized the BCIT X2 RSVP experiment dataset [111,112] and evaluated the quality of generated samples and classification performance using visual inspection (ERP or P300), GMM log-likelihood scores, and AUC metrics. The experimental results demonstrated that the bicubic interpolation combined with deconvolution and bilinear weight initialization (BC-DCBL) method produced the best frequency and amplitude matching for single-channel and multi-channel EEG samples. Moreover, visual inspection showed that the generated samples closely resembled real samples. In the cross-session classification task, the average AUC of CC-WGAN-GP was 82.98 ± 7.68, significantly outperforming EEGNet’s 77.16 ± 13.59.

Aznan et al. utilized neural network-based generative models to create synthetic EEG data to enhance the performance of SSVEP classification [37]. The paper employs three primary neural network generative models: DCGAN and WGAN. The authors trained these models on a limited EEG dataset to generate additional synthetic EEG signal vectors. These vectors aim to augment the dataset used for training the SSVEP classifier. The authors used a CNN-based classification model, which has shown excellent performance in SSVEP classification. The authors used two empirical SSVEP dry-EEG datasets: the Video-Stimuli dataset and the NAO dataset [38]. The generative models were trained on the Video-Stimuli dataset, generating 500 synthetic samples per class, each 3 s long. These synthetic data were then used to improve the classification performance on the NAO dataset. The experimental results demonstrated that the classification performance improved by 3% (DCGAN) and 2% (WGAN) compared to the baseline after adding synthetic data. Furthermore, the authors pre-trained the classifier using only synthetic data and then fine-tuned the model using real data. Compared to the baseline model without pre-training, the pre-trained model showed a significant increase in classification accuracy on the test set, and it was found that using 500 synthetic data samples during the pre-training phase resulted in better average performance of the model. Finally, the authors conducted a cross-subject generalization experiment, evaluating the impact of synthetic data pre-training on the model’s generalization ability by pre-training the model on one subject’s data and testing it on another’s. The results indicated that models pre-trained with synthetic data achieved higher classification accuracy in cross-subject tests compared to models without pre-training.

Kunanbayev et al. explored how to use GANs to generate artificial training data for the classification of P300 event-related potentials in EEG signals [102]. The study mainly investigated the effectiveness of two popular GAN models: DCGAN and WGAN-GP, in generating synthetic EEG data under both subject-specific and subject-independent scenarios. In this study, authors utilized the dataset collected by P. Arico et al. [103]. This study conducted three types of experiments to validate the generative models: GAN testing, t-SNE visualization, and data augmentation classification. In the GAN test, the data from each class of all subjects were pooled to train the generative models, which then generated an equal number of samples. In the data augmentation classification, experiments were conducted separately for subject-specific and subject-independent augmentations, and the classification accuracy was recorded using different amounts of real data. The experimental results showed that the P300 data generated by DCGAN and WGAN-GP could be classified with high accuracy by LDA (Linear Discriminant Analysis) and CNN, and the t-SNE visualization confirmed the high classification accuracy of the generated data. In data augmentation classification, for subject-specific classification, when the training data size was small, the enhancement effect was comparable to that without augmentation, but WGAN-GP performed slightly better, and when the training data size was large, the enhancement effect significantly improved, with WGAN-GP performing slightly better. For subject-independent classification, when the training data size was small, the GAN augmentation effect was better than without augmentation, and when the training data size was large, the GAN augmentation effect was worse than without augmentation, but WGAN-GP performed slightly better than DCGAN.

Guo et al. proposed $R^{2}$WaveGAN, aimed at addressing the issues of shortage and poor quality of EEG [104]. This model is based on the WaveGAN [113] and generates data similar to real EEGs by using a combination of Adaptive Regularization (AR) and Spectral Regularization (SR) as the loss functions. AR is designed to enhance the diversity of the generated EEG signals by calculating the ratio between the input noise and the generative EEG signals. SR aims to improve the similarity between the generated EEG signals and the real EEGs in terms of power spectral density (PSD). The experiments utilized the public dataset Bi2015a [114]. To measure the performance of different models, the authors conducted both quantitative and qualitative analyses. Ultimately, the experimental results demonstrated that $R^{2}$WaveGAN outperformed CC-WGAN-GP in terms of similarity and diversity.

Yin et al. proposed a novel multivariate time series prediction method based on a Multi-Attention Generative Adversarial Network (MAGAN) [105]. The model structure mainly includes three key parts: the encoder network, the generator (decoder) network, and the discriminator network. The primary function of the encoder network is to process the multivariate data’s exogenous sequences and encode them into a latent space, providing input for the generator. It consists of two key attention mechanisms: Input-Attention and Self-Attention. The Input-Attention mechanism dynamically selects the features in the input sequence that are most relevant to the current target, while the Self-Attention mechanism allows the model to capture long-distance dependencies within the input sequence at each time step. The function of the generator (decoder) network is to decode the latent space produced by the encoder network and generate the predicted values of the time series. This process introduces the Temporal-Convolution-Attention module to enhance the model’s ability to capture long-term patterns in the time series. The discriminator network distinguishes between the predicted data and the real data. This study adopted a dynamic weight clipping method to stabilize the training process of the discriminator to improve the overall predictive performance and stability of the model. The authors conducted experimental evaluations on five real datasets, including the EEG dataset from subjects performing the SSVEP experiment [106]. Experimental results show that, in short-term prediction tasks, the MAGAN model outperformed LSTM, Seq2Seq (Sequence-to-Sequence), DARNN (Dual-stage Attention-based Recurrent Neural Network), and TCN (Temporal Convolution Network) across multiple evaluation metrics, especially in terms of MAE and SMAPE, which reached 0.2069 and 0.6635, respectively.

Yin et al. proposed another study for long-term prediction in which they introduced a new model named VAECGAN [107]. This model is also divided into three parts: encoder, generator, and discriminator. In addition to the attention mechanism, both the encoder and generator networks include LSTM. During the generation phase, the output of the attention mechanism is combined with the target sequence, and the prediction values are generated through the LSTM network. The evaluation method and experimental datasets in this study are the same as those in the previous research, but the evaluation metrics are different, using only MAE and Root Mean Square Error (RMSE). The experimental results showed that, compared with LSTM, Seq2Seq, DARNN, TCN, DSTPRNN (dual-stage two-phase attention-based recurrent neural network), and VAE, the VAECGAN model demonstrated superior performance in both MAE and RMSE. Especially on the NASDAQ and SML datasets, the prediction error of the VAECGAN model was significantly lower than that of other models. Additionally, the authors investigated the impact of the dynamic weight clipping threshold on model performance. The results show that appropriately selecting the clipping threshold (e.g., 0.2) can further improve the prediction accuracy.

Xu et al. introduced a novel data augmentation method named BWGAN-GP (Balanced Wasserstein Generative Adversarial Network with Gradient Penalty) aimed at addressing the class imbalance problem in RSVP tasks [108]. BWGAN-GP combines GANs with an autoencoder (AE), where the incorporation of the AE allows both the generator and discriminator to learn common knowledge across all categories. During the GAN’s initialization phase, the generator and discriminator inherit the structure and weights from the trained decoder and encoder. The network structure of BWGAN-GP is inspired by WGAN-GP-RSVP [101], incorporating bicubic interpolation and bilinear weight initialization operations to enhance the quality of the generated EEG data. In the GAN, the discriminator has only one output to avoid the conflict between two outputs (real/fake and category labels) when data are imbalanced. The loss function of the discriminator takes into account the generation of balanced datasets with fake labels, while the generator’s loss function focuses on producing high-quality target class data. The authors evaluated the classification performance of BWGAN-GP-generated data against other methods using a dataset from nine participants, across multiple classifiers, with metrics including AUC, balanced accuracy, F1-score, and Cohen’s kappa coefficient. The results show that BWGAN-GP effectively improves classification performance on deep learning classifiers such as EEGNet, DeepConvNet, and LeNet, with an average AUC value of 94.43% on the EEGNet classifier, which is a 3.7% improvement over the original data.

Kwon et al. introduced a novel signal-to-signal translation method based on StarGAN, designed to convert resting-state EEG signals into specific task-state signals, especially for SSVEP in BCI [109]. This method relies on a multi-domain signal-to-signal translation model named S2S-StarGAN, capable of generating artificial SSVEP signals from short resting-state EEG signals that can serve as individual calibration data. S2S-StarGAN comprises four main components: a generator (*G*), a mapping network (*F*), a style encoder (*E*), and a discriminator (*D*). The generator takes real EEG signals and a style code of the target category as input to produce artificial SSVEP signals of the target category. The mapping network transforms latent codes, sampled from a standard Gaussian distribution, into the target category’s style code. The style encoder (*E*) extracts style codes from real SSVEP signals to guide the signal translation. Lastly, the discriminator judges whether the input samples are real or generated by the generator. Researchers collected EEG data from three subjects for model training and validated the model with data from an additional 15 subjects. The training data were standardized using z-score normalization to compensate for amplitude differences between participants. The results showed that across all test participants, the average classification accuracy using artificial SSVEP signals and the Combined-ECCA method (an extended version of Combined-CCA [115]) reached 95.47%, significantly higher than the 92.03% achieved using the FBCCA (filter bank CCA [116]) method. The experimental results show that the long-duration SSVEP signals generated by TEGAN significantly improve classification accuracy and ITR, especially in scenarios with short time windows and limited calibration data.

Pan et al. proposed a GAN-based end-to-end signal transformation network, called TEGAN (Time Window Length Extension GAN), which is used to transform short-length SSVEP signals into long-length artificial SSVEP signals [110]. TEGAN employs a U-Net generator architecture, combined with a bidirectional Long Short-Term Memory (Bi-LSTM) network, to ensure that the generated signals can capture more spatiotemporal features. To address the issue of mode collapse, TEGAN introduces a LeCam divergence regularization term to constrain the predictions of the discriminator, preventing the generator from memorizing only a few training samples. The effectiveness of the TEGAN model was validated using two public SSVEP datasets: the Direction SSVEP Dataset and the Dial SSVEP Dataset. Experimental results show that TEGAN effectively improves the classification performance of short-length signals by extending the SSVEP signal time window, while significantly reducing the calibration time.

#### 6.1.4. GAN Models for Epilepsy

EEG is a one-dimensional signal in the time domain, used to measure changes in brain electrical activity. It is recorded from electrodes placed on the scalp (EEG) or within the skull (intracranial EEG), to display normal and abnormal conditions such as epileptic seizures [117]. In recent years, with the rapid development of deep learning technologies, algorithms based on deep learning have provided good solutions for the automatic detection and prediction of epileptic seizures. However, these algorithms typically require large amounts of training data, and acquiring EEG signals during epileptic episodes is an expensive and time-consuming process. Consequently, many researchers opt to create synthetic EEG signals that mimic seizures. Among various methods for generating data, GANs are considered a superior method for generating artificial data of epileptic seizures to train epilepsy detection algorithms. Table 8 summarizes the studies of GAN models in the field of epilepsy.

Wei et al. proposed an automatic epilepsy EEG detection method based on CNN [118]. They designed a 12-layer CNN model as the baseline and introduced the Merger of Increasing and Decreasing Sequences (MIDS) and data augmentation methods to enhance the model’s performance. MIDS is a time domain signal processing method aimed at highlighting the characteristic of waveforms. It mainly consists of two steps: merger of clutters and merger of incomplete waves. Through this method, MIDS can highlight key waveform features in the EEG signals, such as spike waves and sharp waves. The researchers used WGAN-GP to generate additional epileptic EEG data, which not only increased the diversity of the training data but also helped to address the class imbalance problem. The dataset used in their experiments was the CHB-MIT Scalp EEG database [119], which includes EEG data of various epilepsy seizure patterns from 23 subjects. The final experimental results showed that the original CNN model achieved 70.68% sensitivity and 92.30% specificity in the epilepsy EEG classification task, while the models with MIDS and data augmentation achieved 74.08% and 72.11% sensitivity, and 92.46% and 95.89% specificity, respectively.

**Table 8:** Studies that used GANs in epilepsy.

Study	Objective	Dataset	GAN Type	Evaluation Metrics	Results
Wei et al., 2019 [118]	Propose a novel automatic epileptic EEG detection method	CHB-MIT Scalp	WGAN-GP	Classification accuracy, sensitivity, specificity	(with respect to raw data) Acc: +0.0351 sen: +0.0143 spec: +0.0359
Pascual et al., 2021 [120]	Generate epileptic EEG signals with privacy-preserving characteristics	EPILEPSIAE	EpilepsyGAN (cWGAN)	Classification accuracy, synthetic data recall geometric mean (sen and spec)	Acc: +1.3% median: +3.2% +1.3%
Usman et al., 2021 [121]	Generate preictal samples to address the class imbalance problem	CHB-MIT Scalp	GAN	Anticipation time, sensitivity, specificity	Average 32 min sen: 93% spec: 92.5%
Salazar et al., 2021 [122]	Oversample the training set of a classifier with extreme data scarcity	Private dataset (Barcelona test)	GAN + vMRF	Probability of error	(with respect to SMOTE) −0.4
Rasheed et al., 2021 [123]	Improve seizure prediction performance	CHB-MIT+ Epilepsyecosystem	DCGAN	Sensitivity, false positive rate, accuracy, specificity	(CHB-MIT) AUC: +6% (Epilepsyecosystem) AUC: +10% Sen: +15%
Xu et al., 2022 [124]	Generate synthetic multi-channel EEG preictal samples	CHB-MIT	DCWGAN	FDRMSE, FID, WD prediction accuracy, AUC	(with respect to DCGAN) FDRMSE: −1.71 FID: −1 WD: −0.21 (with respect to all-real data) Acc: +5.0 AUC: +0.028

Pascual et al. proposed a Generative Adversarial Network named EpilepsyGAN, capable of generating synthetic epileptic EEG signals with privacy-preserving characteristics [120]. EpilepsyGAN adopts the architecture of a conditional GAN, comprising a generator and a discriminator, where the generator adopts a U-net convolutional autoencoder structure, with interictal signals as input and ictal signals as output. To increase the diversity of samples, Gaussian noise is added to the output of the encoder. For training EpilepsyGAN, the researchers utilized data from the EPILEPSIAE project database [125]. During evaluation, the authors used a classifier based on the random forest algorithm, whose primary task is to distinguish whether EEG signals are in the seizure (ictal) or non-seizure (interictal) phase. The final experimental results indicated that, through visual inspection and FFT similarity analysis, the synthetic signals were similar to real epileptic signals. Furthermore, compared to systems trained with real epileptic samples from a general database, the epilepsy detection system trained with synthetic samples achieved a 1.3% improvement in performance. For 67% of patients, the system trained with synthetic data showed more than a 1% increase in performance compared to the system trained with real data.

Usman et al. proposed a method to predict the preictal state, which precedes epileptic seizures, by monitoring brain activity through EEG signals [121]. This method is divided into three main steps: preprocessing, feature extraction, and classification. In the preprocessing phase, the authors utilized Empirical Mode Decomposition (EMD) to remove noise from the EEG signals and GAN to address the class imbalance issue. This approach increases the number of preictal state samples in the dataset, helping to resolve the class imbalance problem. The researchers then employed a CNN to automatically extract features from the EEG signals. This CNN architecture includes three convolutional layers, each followed by an activation layer, a pooling layer, and batch normalization. Finally, LSTM units are used to differentiate between the preictal and interictal states based on the extracted features. The dataset used for the experiment was the publicly available CHBMIT scalp EEG dataset, which contains EEG records from 22 patients with epilepsy. The experimental results show that this method can predict epileptic seizures with 93% sensitivity and 92.5% specificity, on average 32 min in advance.

Salazar et al. combined two fundamental concepts: GAN and vector Markov Random Fields (vMRFs), to propose a novel approach (GANSO) for oversampling very small training sets [122]. vMRF is an extension of the classical MRF, which is a generalized Markov Random Field that assumes vectors instead of scalars are assigned to the graph vertices. The generative module of the GAN model randomizes in the frequency domain through the Graph Fourier Transform (GFT) and then generates synthetic instances by transforming back to the original domain via the inverse Fourier transform. Subsequently, the discriminative module uses a linear discriminant function to distinguish between original instances and synthetic instances. These two modules continuously compete through iterative optimization. The authors validated their method using three datasets (EEG [126], fMRI, ECG). The experimental results show that, compared to SMOTE (Synthetic Minority Oversampling Technique), the synthetic instances generated by GANSO significantly reduce the classifier error rate, while SMOTE performs poorly in scenarios with such a small amount of data.

Rasheed et al. proposed a method based on DCGAN to generate EEG data for epileptic seizure prediction [123]. The authors utilized two datasets: the CHBMIT dataset [119] and the Epilepsyecosystem dataset [127]. Initially, EEG signals were transformed into spectrograms using short-time Fourier transform (STFT) to be suitable for CNN models. Subsequently, DCGAN was employed to generate synthetic iEEG and scalp EEG data. The generator accepted random samples from a standard Gaussian distribution as input and produced outputs with dimensions identical to real EEG spectrograms through multiple layers of deconvolution networks. The discriminator’s task was to differentiate between real and generated spectrograms. To assess the quality of the synthetic data, the authors utilized a one-class SVM and a convolutional epileptic seizure predictor (CESP). The CESP model exhibited sensitivities of 78.11% and 88.21% and FPR/h of 0.27 and 0.14, respectively, on synthetic training and testing on real Epilepsyecosystem and CHB-MIT datasets. These results surpassed those obtained by training and testing the CESP model on real data. After validating synthetic data, the authors evaluated the performance of four renowned deep learning models (VGG16, VGG19, ResNet50, and Inceptionv3) on the task of epileptic seizure prediction through transfer learning. These models were first pre-trained on the augmented dataset and then fine-tuned for specific patients to develop personalized seizure prediction algorithms. The final experimental results indicated that Inceptionv3 and ResNet50 performed better, especially Inceptionv3, which achieved a sensitivity of 90.03% and an FPR/h (false positive rate per hour) of 0.03 in the epileptic seizure prediction task.

Xu et al. proposed a preictal artificial signal synthesis algorithm based on GAN to generate synthetic multi-channel EEG preictal samples, addressing the issue of insufficient annotated EEG signals required for training deep learning algorithms [124]. The study considered GAN models based on CNN and RNN for generating single-channel EEG preictal samples. CNN models are suitable for processing image data, capturing spatial features through stacked convolutional layers. On the other hand, RNNs, especially LSTM, are suitable for handling time series data, capturing temporal dependencies. After selecting the best-performing single-channel generation model (DCWGAN), the researchers independently trained generators for each EEG channel. Then, they used the same noise vector as input to generate single-channel samples from all trained generators and combined these single-channel samples into complete multi-channel preictal EEG samples. Based on the CHB-MIT EEG dataset, the authors evaluated the effectiveness of synthetic samples by comparing the performance of epileptic seizure prediction under non-augmented and augmented conditions, using accuracy and AUC as evaluation metrics. The study results showed that after augmenting data with synthetic preictal samples, the accuracy improved by approximately 5% on average, and the AUC improved by approximately 3% on average.

#### 6.1.5. GAN Models for Other EEG Applications

The previous sections primarily discussed the application of GAN models in the five main paradigms based on EEG. This section includes additional studies on using GAN models to generate EEG data, summarized in Table 9.

Yao et al. proposed using a GAN-based autoencoder to filter out unwanted features from EEG signals [128]. The model they introduced is structured similarly to CycleGAN [138], but specifically tailored for filtering features in EEG signals. Initially, EEG signals are converted into images by applying a Fast Fourier Transform (FFT) to extract the energy of different frequency bands and mapping this information onto images, thus creating color images that represent the original EEG signals. During the training process, the generator tries to generate new EEG signal images devoid of unwanted features (e.g., alcohol information) while preserving important information as much as possible (e.g., stimulus response information). The training process consists of two cycles, each with two types of loss: adversarial loss and reconstruction loss. The experiments utilized the UCI EEG dataset, which includes EEG signals from subjects with alcoholism and control groups [139]. To evaluate the model’s performance, an additional classifier was employed to measure the change in classification accuracy of the generated EEG signal images after removing specific features. The results showed that after processing with the GAN-based autoencoder, the proportion of original images correctly classified as containing alcohol information dropped from 96.1% to 29.8%, indicating that the model effectively filtered out a large portion (about 66.3%) of alcohol information. At the same time, the accuracy of stimulus information classification only dropped by 6.2%, still significantly above chance level, which is 20% in this study as there are five stimulus conditions.

Hazra et al. introduced SynSigGAN for generating various types of biomedical signals [129]. This model utilizes a bidirectional grid Long Short-Term Memory (BiGridLSTM) network as the generator and a CNN as the discriminator. LSTM is capable of addressing the long-term dependency issue found in traditional RNNs, allowing for the retention of information over longer periods. BiGridLSTM further enhances this by integrating GridLSTM bidirectionally, enabling it to simultaneously capture contextual information and reduce the vanishing gradient problem across two dimensions (time and depth). During the data preprocessing stage, the authors applied wavelet denoising to process biomedical signals and used a Daubechies wavelet filter to process the raw signals. Then, they applied threshold processing to the outputs of the high-pass and low-pass filters and finally produced the denoised signal through inverse discrete wavelet transform (IDWT). This study involves four types of biomedical signals: ECG, EEG, EMG, and photoplethysmography (PPG). The model’s performance is evaluated using RMSE, MAE, Percent Root Mean Square Difference (PRD), Fréchet Distance (FD), and Pearson’s correlation coefficient (PCC). The experimental results showed very high Pearson correlation coefficients between the synthetic and original data across all four types of biomedical signals, with the EEG signal reaching a correlation coefficient of 0.997. Additionally, the synthetic signals exhibit relatively low values for RMSE, PRD, and MAE, with the EEG signal having an RMSE of 0.0314, PRD of 5.985, and MAE of 0.0475, indicating the high quality of the synthetic signals. Furthermore, when compared to existing synthetic signal generation models such as BiLSTM-GRU and BiLSTM-CNN GAN, SynSigGAN demonstrates superiority across all evaluation metrics.

Tazrin et al. proposed a framework called LiHEA (Logic-in-Headbands-based Edge Analytics), which aims to migrate EEG data analysis from cloud computing to ultra-edge devices closer to users, such as EEG headbands [130]. The model is a lightweight AI model, and researchers used the Min-Max Scaling method to normalize data to handle features of varying magnitudes. To enhance the training data and improve the generalizability of the model, researchers utilized Deep DCGAN to generate EEG data. The LiHEA framework was tested through multiple experiments to assess the effectiveness of different models and methods in EEG data analysis. The experiments evaluated the performance of various traditional machine learning and deep learning models, including Random Forest, SVM, KNN, Logistic Regression, DNN, and CNN. The main comparison metric was classification accuracy. The experimental results showed that among all models evaluated, Random Forest performed the best, achieving an accuracy of up to 90%. Furthermore, the training of models was enhanced by synthetic EEG data generated through DCGAN, significantly improving the performance of DNN and CNN. Particularly, when the generated data volume was 800% of the original data, the models performed best.

Krishna et al. utilized an RNN-based regression model and GANs to predict EEG features from acoustic features [131]. The GAN model consists of a generator and a discriminator, where the generator model comprises two layers of Bi-GRU with different numbers of hidden units, followed by a time-distributed dense layer. The objective of the generator is to generate data from a latent space, meaning it takes mel-frequency cepstral coefficient (MFCC) features as input and outputs a vector with dimensions matching the EEG feature set. The discriminator consists of two single-layer Bi-GRUs connected in parallel and running concurrently. The experimental results primarily focused on the effectiveness of using RNNs (particularly GRU and Bi-GRU models) and GANs to predict EEG features from acoustic features. These experiments were conducted on two datasets: Dataset A (simultaneous voice and EEG recordings from four subjects) [140] and Dataset B [141]. The results showed that, regardless of whether GRU or Bi-GRU models were used, the RMSE from predicting EEG features from acoustic features (listening MFCC) remained relatively consistent among all subjects. The experiments on Dataset B did not differentiate among individual subjects. Instead, the entire dataset was used for training, validation, and testing. The results of using GRU models to predict EEG features also demonstrated lower average RMSE values, indicating that this method has generalizability across different datasets. Although GANs could generate more accurate EEG features by learning the data distribution in theory, the experimental results showed that GRU and Bi-GRU models outperformed GANs in prediction accuracy.

Lee et al. proposed SIG-GAN (Signal Generative Adversarial Networks) to fill in missing data in EEG signal sequences [132]. The SIG-GAN model is based on the GAN framework, where the generator employs an autoencoder structure. It captures high-frequency and low-frequency features through two parallel CNN layers, ultimately outputting the generated EEG signal segments. The discriminator also adopts a CNN architecture, effectively extracting features from the EEG signals. Additionally, the model introduces an auxiliary classifier to assist in generating signals that retain important medical contextual information, such as sleep stages. The data processed by the model are EEG signal sequences, each containing multiple 30-second-long signal segments, with each segment having a corresponding sleep stage label. Data preprocessing includes normalizing the EEG signals to the range of [−1,1]. Training the SIG-GAN model involves optimizing multiple loss functions: adversarial loss, reconstruction loss, and prediction loss. By inputting the generated EEG signals into existing automatic sleep stage scoring models (DeepSleepNet [142] and SleepEEGNet [143]), the paper evaluates the usability of the signals after missing data have been filled in. Experimental results show that even with up to 50% missing data, the EEG signals filled in using SIG-GAN can still be accurately classified by these sleep stage scoring models. In addition, the signals generated by SIGGAN are significantly superior in quality and diversity compared to RANDOM (which imputes missing signals by independently sampling random values within the range of −1 to 1) and EEGGAN, as evidenced by the higher IS (2.41) and the lower FID (16.83).

An et al. proposed a WGAN-based denoising method that can automatically remove noise from multi-channel EEG signals [133]. Before inputting EEG data into the GAN model, the authors performed preprocessing steps such as frequency filtering, re-reference, and baseline removal. Then, they proposed a sample entropy threshold and energy threshold-based (SETET) normalization method to normalize the EEG signals, ensuring that the signals input into the GAN model are within an appropriate range. During the training process, the authors first input the noisy EEG data into the generator, which tries to output the denoised data. Then, both the generated data and the real clean data are input into the discriminator. The discriminator needs to learn to distinguish between the generated data and the real clean data. The experiments utilized the publicly available HaLT dataset [49], which contains EEG data from 12 subjects, with approximately 950 trials per subject, and each trial records data from 22 channels. The authors used correlation and RMSE as the main indicators for assessing the denoising performance. They conducted two sets of experiments to evaluate the performance of the proposed WGAN-based EEG signal denoising method. First, they tested the denoising performance of the model after training on data from a single subject. The results showed that the denoised EEG data for most subjects had a high correlation with the original data (average correlation of 0.7723) and a relatively low RMSE (average of 0.0792). Next, they trained the model on data from multiple subjects and then tested it on data from subjects who were not involved in the training. The results showed that when the model was trained on data from multiple subjects, the denoising effect exhibited better generalization capability, with the average correlation increasing to 0.7771 and the RMSE decreasing to 0.0757.

Sawangjai et al. introduced a framework named EEGANet, which is based on GANs to address the problem of removing ocular artifacts from EEG signals [134]. EEGANet adopts a calibration-free approach that does not require additional EOG channels or eye-blink detection algorithms, allowing for the direct application of the model to remove ocular artifacts after training. EEGANet was tested on three different datasets: the EEG Eye Artifact Dataset, BCI Competition IV 2b Dataset, and Multimodal Signal Dataset. For each dataset, EEGANet performed a series of preprocessing steps, including filtering, labeling, and normalization, to prepare the data for model training. Experimental results showed that the average RMSE of the generated signals by EEGANet is lower in both time and frequency domains. Moreover, by comparing the RMSE under different SNRs, the results demonstrate that EEGANet maintains good performance when dealing with EEG signals with varying degrees of noise. Furthermore, validation on the BCI Competition IV 2b Dataset revealed that EEGANet could improve classification accuracy and F1-scores for the left- and right-hand motor imagery task compared to the original signals. Validation on the Multimodal Signal Dataset showed that EEGANet-processed signals are effectively used for classifying rest states versus motor imagery/execution states, displaying good performance compared to traditional methods that require EOG channels.

Abdi-Sargezeh et al. proposed a model named VAE-cGAN, which combines cGAN and VAE for mapping scalp electroencephalography (sEEG) to intracranial electroencephalography (iEEG) [135]. The model aims to enhance the resolution of sEEG, thereby improving the detection of interictal epileptiform discharges (IEDs) in clinical practice. The VAE-cGAN consists of three main components: an encoder, a generator, and a discriminator. The encoder maps the input sEEG data to a latent space *z*. The generator, receiving the encoded latent space *z* and sEEG data as input, generates an estimate of iEEG. The generator is designed based on spatially adaptive normalization blocks (SPADE). SPADE is a conditional normalization technique that denormalizes layer activations based on external data. Then, the authors used a Markovian discriminator (patch discriminator) [144] to differentiate between the generated iEEG data and real iEEG data. The training of VAE-cGAN is accomplished by combining the Generator’s hinge loss, KL divergence loss, L1 loss, and feature matching loss. The dataset used in the experiment comes from 18 epilepsy patients at King’s College Hospital London. The results indicate that the VAE-cGAN method performs excellently in both inter-subject and intra-subject classification approaches. The average accuracy for inter-subject classification is 76%, which is an improvement of 11%, 8%, and 3% over the previously proposed least-square regression (LSR), asymmetric autoencoder (AAE), and asymmetric–symmetric autoencoder (ASAE) methods, respectively. The average accuracy for intra-subject classification is 69%, which is an improvement of 7%, 3%, and 1% over the LSR, AAE, and ASAE methods, respectively. Furthermore, the VAE-cGAN outperforms other methods in terms of sensitivity and specificity, achieving 68% and 70%, respectively.

Yin et al. proposed a GAN-guided parallel CNN and Transformer network (GCTNet) for EEG denoising [136]. The GCTNet model consists of two main parts: a generator (*G*) and a discriminator (*D*). The design of the generator integrates both CNN and Transformer structures to capture the local and global temporal dependencies of EEG signals, effectively removing noise. The discriminator aims to distinguish between real clean EEG signals and denoised EEG signals generated by the generator, thus providing additional loss functions to guide the training of the generator. The loss functions of the generator used in model training include MSE loss, feature loss, and adversarial loss. The denoising performance of GCTNet is demonstrated by comparison with other methods on various datasets, including EEGDenoiseNet [145], the MIT-BIH Arrhythmia Dataset [146], a semi-simulated EEG/EOG dataset [147], and the BioSource database. Multiple evaluation metrics quantify the denoising performance, including relative root mean squared error (RRMSE), correlation coefficient (CC), signal-to-noise ratio (SNR), weighted signal-to-noise ratio (WSNR), and weighted correlation coefficient (WCC). The results show that GCTNet outperforms existing state-of-the-art methods in noise removal tasks on semi-simulated datasets. On real data, GCTNet effectively reduces interference caused by muscle activity and eye movements while preserving the key features of the original EEG signals.

Wickramaratne et al. introduced a system based on cGAN capable of generating EEG signals during non-rapid-eye-movement sleep [137]. The cGAN’s generator inputs the random noise vector and conditional information (sleep stage labels) into a series of one-dimensional convolutional layers and deconvolutional layers. These layers gradually upsample the signal’s length, ultimately generating synthetic EEG signals that resemble real EEG signals. The experiment utilized polysomnography (PSG) data from healthy subjects in the Multi-Ethnic Study of Atherosclerosis (MESA) [148], which were obtained through the National Sleep Research Resource (NSRR). The authors compared the characteristics of the synthetic signals with real EEG signals, paying particular attention to the relative power percentages in different frequency bands (α, β, θ, δ, and σ). The results indicated that the cGAN-generated EEG signals visually resemble real signals, especially in depicting sleep features of stages 2 and 3, such as K-complexes and slow waves. However, there are notable differences between CGAN-generated and real EEG signals in certain frequency bands, particularly in the θ and σ bands.

### 6.2. Conclusions

This section provides an overview of the current research status of GAN models used for EEG generation, elaborating on their exceptional contributions to addressing EEG data scarcity from five aspects: motor imagery, emotion recognition, external stimulation, epilepsy seizure detection and prediction, and other applications. Compared to the VAE models summarized in the previous section, GANs have made significant improvements in the quality of generated data. However, training GAN models is not easy, often requiring longer time and being unstable, with issues such as mode collapse. Additionally, evaluating the quality of generated data has always been a challenge for GAN models. Although current research assesses model performance using metrics such as accuracy and F1-score, the subjective evaluation of generated data remains challenging.

## 7. Diffusion Models for EEG

In recent years, diffusion models have emerged as a powerful class of generative models, achieving remarkable success in image, audio, and text generation tasks. Compared to GANs and VAEs, diffusion models offer more stable training and are capable of generating high-quality synthetic data. These advantages have gradually attracted attention in the field of EEG research, particularly for addressing challenges related to limited data availability and improving the quality of generated signals. This chapter provides an overview of recent applications of diffusion models in EEG data generation, with a focus on their model architectures and the performance achieved across various brain–computer interface tasks.

### 7.1. Review of Related Work

In this section, we reviewed the various applications of diffusion models in EEG tasks, and Table 10 summarize all the articles on the use of diffusion models across various EEG tasks.

Tosato et al. discussed the method of generating synthetic EEG data using the denoising diffusion probabilistic model (DDPM) in their study [149]. Notably, the data generated by DDPM in this paper are EFDMs (electrode-frequency distribution maps [163]) rather than the EEG data themselves. The creation of EFDMs involves preprocessing of EEG data, where the raw data are read using the MNE-Python library and converted into two-dimensional matrices. Subsequently, the short-time Fourier transform (STFT) is applied to these data, producing EFDMs as grayscale images that display the intensity values of each frequency for every channel. In the experiments, a classifier was trained on real EEG data firstly, and then the same classifier was used to evaluate the synthetic data generated by DDPM. The experimental data were derived from EEG data of specific emotions (happiness and sadness), with 24,000 images used for each emotion. The final results of the experiments indicate that the “hybrid” model, which was trained using a combination of synthetic and real data, exhibited better performance than the model trained only with real data. The average accuracy was 92.63% when using data generated by DDPM after 40 training epochs, and 92.98% after 60 training epochs.

Shu et al. introduced a method named DiffEEG, based on diffusion models, aimed at enhancing data for the prediction of epileptic seizures [150]. DiffEEG is trained using the preictal EEG signals of patients, with the goal of generating sufficient samples to balance the number of samples in each category. The generated samples are then used for the task of predicting epileptic seizures. Experimental results show that the DiffEEG method significantly outperforms other data augmentation methods, such as undersampling, sliding window, and recombination, in terms of performance on seizure prediction tasks. By evaluating with multi-layer perceptrons (MLPs), multi-scale CNNs, and transformers as classifiers on the CHB-MIT and Kaggle databases, DiffEEG achieved higher sensitivity, lower false positive rates (FPRs), and higher AUC values across all three classifiers.

Aristimunha et al. utilized latent diffusion models (LDMs) with spectral loss to generate 30-s sleep stage EEG signals [151]. Firstly, they extract samples from the sleep EEG dataset and select a 30-s window for each electrode. These 30-s windows are then used individually and combined with data from other subjects to create the dataset. A trained autoencoder is used to map the input data to a latent space representation *z*, which is then used for data generation. In the autoencoder, the authors employ KL regularization to prevent the network from simply copying the input to the output, instead forcing it to learn a meaningful representation of the input data. After training the LDM, the decoder with an attention-based residual U-Net transformed the compressed representation *z* back into EEG signals. The authors trained the model using two large sleep datasets (Sleep EDFx [164] and SHHS [165]) and evaluated the quality of the generated EEG signals through the Multi-Scale Structural Similarity Metric (MS-SSIM), FID, and PSD within the sleep band. The final experimental results demonstrated that the LDM with spectral loss (LDMspec) showed better results in the FID and MS-SSIM metrics, indicating that the signals generated by these models are closer to real signals. Specifically, the MS-SSIM values for LDMspec in the Sleep EDFx and SHHSh datasets were 0.515 and 0.598, respectively, while the values for the LDM without spectral loss were 0.308 and 0.168. Additionally, from the PSD analysis, it can be observed that the LDM model with spectral loss better captures the structural oscillations in the signal, especially in the δ band.

Sharma et al. introduced a method called MEDiC to mitigate the issue of EEG data scarcity by using a class-conditioned DDPM [152]. The authors began by segmenting EEG signals into 30-s segments with a 25% overlap and used EEGNet as a semantic encoding network to extract 784-dimensional latent embeddings from these segments. Subsequently, they developed a class-conditioned DDPM trained for these latent EEG embeddings. Upon successful training, 500 synthetic samples were generated for each class. When training a multi-layer perceptron (MLP) with the synthetic embeddings and testing it on unseen original embeddings, robust classification results were observed for AD-CN (Alzheimer’s Disease vs. Cognitive Normal) and FTD-CN (Frontotemporal Dementia vs. Cognitive Normal) cases. This indicates that the synthetic EEG embeddings generated through DDPM preserved key category-distinguishing information essential for downstream tasks. Moreover, the synthetic data exhibited lower divergence from the original data in terms of the Jensen–Shannon divergence (JSD) score. The final experimental results showed that the DDPM-generated synthetic embeddings consistently demonstrated higher accuracy, recall, and F1-scores in AD-CN and FTD-CN classifications compared to embeddings generated through VAE.

Torma et al. introduced a novel DDPM named EEGWave, for the generation of EEG signals [153]. The EEGWave model maintains the same dimension for both input and output data, omitting sampling layers to avoid temporal and spectral artifacts during the data synthesis process. The model comprises multiple residual layers, each connected to an input and output stream. Within the residual layers, encoded and embedded diffusion noise is added to the input’s temporal features, and then the data are processed through a bidirectional dilated convolution layer. Throughout the training process, the model learns how to denoise from initial white noise samples gradually, generating synthetic event-related potential (ERP) epochs. Finally, the authors compared the performance of EEGWave with WGAN in generating EEG signals, utilizing evaluation metrics such as IS, FID, and SWD. Experimental results showed that EEGWave surpassed WGAN across all three evaluation metrics, demonstrating higher signal fidelity and diversity.

In another study from the same year, the authors proposed a method based on diffusion probabilistic models (DPMs) for generating high-quality EEG signals, specifically visual evoked potentials (VEPs) [154]. To reduce the number of sampling steps in the generation process and enhance efficiency, the authors adopted a progressive refinement technique. This approach incrementally decreases the number of generation steps through multiple training stages, where each stage trains a new student model to mimic the output of a teacher model, but with fewer steps to accomplish the same task. Furthermore, the authors designed a special neural network architecture without upsampling and downsampling layers to avoid spectral artifacts in the generation of EEG signals. This architecture utilized bidirectional dilated convolutions to maintain the global context and consistency of temporal data. The experiments were conducted using the publicly available VEPESS dataset [166], which contains visual evoked potential recordings from 18 subjects. Quantitative metrics included SWD, Gaussian Mixture Model Score Difference (dGMM), IS, and FID. The results indicated that the generated EEG signals were of a quality similar to the original dataset, both through direct and indirect metrics. Moreover, the authors used Kolmogorov–Smirnov, Mann–Whitney U, and Kruskal–Wallis tests to compare the distributions of original and generated samples. The test results showed that the generated signals were able to maintain distribution characteristics similar to the original signals, especially in the in-class case.

Soingern et al. demonstrated the use of a diffusion model based on WaveGrad as a data augmentation (DA) method for motor imagery classification in [155]. The author applied DA using five standard EEG motor imagery models (EEGNet, ATCNet, EEG-ITNet, Deep ConvNet, and ShallowFBCSPNet) on synthetic data of various sizes from the BCI Competition IV 2a to evaluate the effectiveness of the proposed method. The experimental results indicated that the proposed method enhanced performance, surpassing other traditional EEG data augmentation methods. WaveGrad is a gradient-based text-to-speech synthesis model that employs diffusion probabilistic models for voice generation. The core idea of this model is to recover clear speech signals from noisy data through a gradual process of noise removal.

Zhou et al. proposed an EEG data augmentation framework called Diff-EEG, based on diffusion models, designed to enhance the detection of Alzheimer’s Disease (AD) [156]. This framework comprises three modules: a Continuous Wavelet Transform (CWT) module, a spatial VQ-VAE module, and a guided diffusion model module. Initially, EEG signals are transformed from the time domain to the time–frequency domain using CWT, obtaining the time–frequency spectra of multi-channel EEG data. Subsequently, the VQ-VAE takes the multi-channel time–frequency spectra as input, learning a compact and information-rich low-dimensional representation (referred to as “spatial code”), which captures the spatial, temporal, and frequency domain features of the input EEG data. Finally, the guided diffusion model architecture, taking the spatial code as input, leverages subject labels to guide the model in generating class-specific EEG latent variables. In the experimental section, the authors utilized an open-source dataset from [167], assessing the quality of the augmented data by incorporating them into the original dataset and evaluating them based on the classifier’s performance. EEG-Net was used as the classifier for the AD diagnosis task, with performance metrics including accuracy, precision, and recall. The experimental results showed that Diff-EEG achieved higher accuracy, precision, and recall compared to other data augmentation methods (such as adding Gaussian noise, VAE, DCGAN, VAE-GAN) across different data augmentation scales. Specifically, at data augmentation scales of two and three, Diff-EEG achieved accuracy rates of 89.3% and 93.3%, respectively.

Vetter et al. explored the application of DDPMs for generating realistic neuro-physiological time series in [157]. They conducted multiple experiments to validate the performance of DDPM across various datasets, including different recording techniques (LFP, ECoG, EEG) and species (rats, macaques, humans). The experimental results indicated that DDPMs are capable of generating synthetic data that are consistent with real recordings in both single-channel and multi-channel statistical properties. Moreover, the data generated by DDPM significantly improved the performance of neural decoding tasks by enhancing the interpolation of missing data.

Wang et al. proposed a deep learning framework based on diffusion models, named DiffMDD, for diagnosing Major Depressive Disorder (MDD) using EEG data [158]. The DiffMDD framework comprises three parts. Firstly, a forward diffusion noise training module injects Gaussian noise into the original EEG data to help the model learn features that are unrelated to noise, thereby enhancing the model’s robustness. Secondly, a reverse diffusion data augmentation module aims to address the issue of data sparsity by generating new EEG samples. To improve the quality and diversity of the generated data, this module utilizes the output gradients and EEG embeddings of a classifier preliminarily trained in the forward diffusion noise training module as guidance and condition. Lastly, the classifier is retrained using the augmented EEG dataset to obtain the MDD diagnosis results. The framework was validated on two publicly available MDD diagnosis datasets and achieved outstanding performance. Specifically, the DiffMDD model achieved state-of-the-art performance on all evaluation metrics, including sample-level and subject-level accuracy, as well as F1-scores.

Wang et al. explored how to protect the intellectual property (IP) of EEG-based models and proposed a method named DeRiF (Diffusion and Retrieval Fingerprinting) in [161]. This method primarily includes two stages: fingerprint validation set construction and model fingerprint matching. In the fingerprint validation set construction stage, the authors designed a Conditional Denoising Diffusion Probabilistic Model (CDDPM) to generate high-quality and diverse simulated EEG samples. To effectively process EEG signals, CDDPM employs a Conditional UNet architecture adapted for EEG signal processing. The UNet consists of two main paths: the contracting path and the expansive path. In the contracting path, the input signal $e\in R^{32\times 128}$ undergoes a series of convolutional and max-pooling operations, progressively compressing the spatial dimensions of the signal while increasing the depth of the feature representation. In the expansive path, the spatial dimensions of the signal are gradually restored through upsampling and convolutional operations. To validate the effectiveness of CDDPM, the authors used the DEAP dataset and conducted experiments under various attack scenarios. Through PCA visualization and plotting EEG topographic maps, they demonstrated that CDDPM can generate samples with feature distributions highly similar to real data. Moreover, the generated samples show activation patterns in different brain regions that are very similar to those of real data, while also exhibiting a certain degree of diversity.

Klein et al. proposed a new conditional diffusion model to address the issue of data scarcity in the brain–computer interface (BCI) field, particularly the common class imbalance problem in ERP [162]. The diffusion model used by the authors is based on the “Variance Preserving Stochastic Differential Equation” (VP-SDE), which is the continuous form of DDPM. The model architecture is similar to EEGWave and diff-EEG, which have been shown to perform well in handling EEG data. However, there are two key improvements in this paper. First, the time step embedding is rewritten to be compatible with VP-SDE. Second, the authors do not normalize the EEG data to preserve its original features. During the training process, the authors adopted classifier-free guidance by introducing additional conditions (such as subject, session, and class) into the model without relying on a classifier for guidance. The authors used the visual ERP dataset from Lee et al. [168] and evaluated the effectiveness of the proposed model in generating EEG data through a series of experiments. The experimental results showed that the highest averaged balanced accuracy (ABA) for generated data was 0.817, which is very close to the 0.818 for real data. In some subject and session combinations, the classification performance of the generated data even exceeded that of the real data. Furthermore, the SWD value of the generated data was 1.02, lower than the SWD (1.46) between different sessions of the real data. Additionally, the standard deviation Manhattan distance (SD-MD) values of the generated data for target and non-target data were 3.44 and 1.33, respectively, both lower than the SD-MD (5.16) between different sessions of the real data. The peak amplitude delta (PAD) value of the generated data was 0.48 μV, lower than the PAD (0.83 μV) between different sessions of the real data, indicating that the generated data performed better in terms of P300 peak amplitude compared to the real data between different sessions.

### 7.2. Conclusions

This section provides an overview of the current applications of diffusion models for EEG generation, emphasizing their contributions to enhancing EEG data. Compared to the previously summarized models (VAEs and GANs) and traditional data augmentation methods, diffusion models offer unique advantages. For instance, the EEG data generated by diffusion models contain fewer artifacts, are of higher quality, and are more diverse, performing exceptionally well in many specific tasks. For example, diffusion models excel at synthesizing EEG signals with specific frequency band structures (such as δ, θ, and α waves), accurately capturing both single-channel and multi-channel statistical data, such as frequency spectra and phase–amplitude coupling, as well as dataset-specific features. Due to the randomness in their generation process, diffusion models better cover the latent space of the data, producing more diverse samples. Compared to GANs, diffusion models have theoretical advantages in capturing the dynamic changes in signal structure, better preserving the structural information of EEG signals. Additionally, the training of diffusion models is more stable and does not require special loss functions, avoiding the issue of mode collapse during training, which ensures the diversity of the generated data. Considering these advantages, current data augmentation methods using diffusion models perform best in data augmentation. Despite the significant improvements of diffusion models over the previous two generative models, there are still some issues that need to be addressed. The most apparent problem is that diffusion models involve many sampling steps, resulting in slow inference speed. The high computational complexity during training and generation requires substantial computational resources and time.

## 8. Summary and Outlook

This review provides an overview of three mainstream generative models (VAE, GAN, diffusion models) and their various applications in EEG generation, revealing their techniques, model architectures, achievements, and challenges. Despite the excellent performance of generative models in addressing the scarcity and limitations of small-scale EEG datasets in various EEG tasks, several challenges remain. For example, the data generated by VAE models often lack quality, GANs are prone to mode collapse during training, and diffusion models have long inference times. Overcoming these challenges is essential for progressing the field and unlocking the full potential of generative models in EEG generation.

Based on the above summary, we believe that future research will focus on the following directions:

- Ensuring the validity of generated data is an important issue. Most current research uses metrics like FID, JS divergence, MMD, and IS to evaluate the similarity between generated data and real data. However, to date, there has not been a direct metric that can be considered a true assessment of the quality of generated EEG signals, nor one that directly correlates with model performance. Therefore, designing better evaluation metrics is a problem that needs to be addressed. Future research should also focus on how to extract more representative features from generated EEG signals and establish boundary conditions for the feature distribution of the generated data.
- From lab to real world. Although various generative models have made significant progress in the field of BCI in recent years, the vast majority of studies still rely on datasets collected under highly controlled laboratory conditions. According to the review by Altaheri et al. [9], EEG signals are inherently noisy, non-stationary, and highly susceptible to individual differences and environmental influences. Laboratory-collected data typically contain fewer physiological artifacts and less environmental interference, which does not accurately reflect the complexity of real-world applications. Moreover, although many deep learning models claim to work with raw EEG signals, in practice, they still heavily depend on preprocessed data, such as artifact removal and band-pass filtering, limiting their adaptability in unstructured environments. Therefore, designing robust MI-BCI systems that can operate under real-world conditions, which are characterized by high noise, dynamic variability, and low signal-to-noise ratios, remains a critical and unresolved challenge. Future research should place greater emphasis on collecting EEG data in naturalistic settings and developing models with strong generalization capabilities to enhance their practicality and reliability in real-world applications.
- The impact of individual differences. There are significant differences in the EEG signals of each subject, and current research mostly focuses on complex network architecture designs while neglecting the issue of target data distribution differences. Therefore, in the future, an effective model that encompasses a wide range of subject variations and can handle data from different subjects could be developed. Some existing studies have already employed unsupervised end-to-end inter-subject transfer methods to address the inter-subject problem [74].
- The incorporation of other modalities. Some other biological signals, such as ECG or EMG, share similar characteristics with EEG signals, including non-linearity, non-stationarity, and temporal correlation. Current research has successfully transformed EEG signals into fMRI signals using innovative multi-dimensional feature extraction and deep learning methods [168]. Future studies could consider combining EEG signals with other biological signals (such as eye movement data, electromyography signals) or non-biological signals (such as images or speech) for further improvements.
- The strategy of using generated data. Currently, some studies have explored the impact of varying proportions of generated data on performance and have demonstrated that the effect of data augmentation is not a linear relationship with the amount of generated data [65]. Therefore, determining the optimal amount of data to generate for the most effective enhancement of classifier performance is a direction for future exploration.
- Hybrid models. The three models outlined in this paper are popular deep learning generative models, each with unique features and suitable for different application scenarios. Each model has its strengths and weaknesses. In future research, combining these models could be considered, such as combining VAE and diffusion models to perform diffusion in a low-dimensional space, thus improving computational efficiency and capturing the low-dimensional representations of neuro-physiological recordings. This approach could leverage the strengths of various generative models and further enhance the quality and diversity of synthetic data. Current research has successfully combined cGAN with VAE to translate scalp EEG signals into higher quality intracranial EEG [135].
- Model interpretability. VAE, GAN, and diffusion models are all data-driven, generating new data by learning from data. These models do not directly rely on an understanding of the system’s intrinsic physical or biological mechanisms but depend on large amounts of data to train the networks, enabling them to generate realistic data samples. Future research should focus on improving the interpretability of these models to better understand the decision-making processes and the characteristics of the data they generate. Some studies have already employed Layer-Wise Relevance Propagation (LRP) techniques to explain the decision-making processes of these models [135].
- The integration of large models. The emergence of large-scale EEG foundation models such as LaBraM [169], CBraMod [170] and NeuroLM [171] has not only advanced representation learning and multi-task transfer but also offered new inspiration for EEG data augmentation. These models, trained on massive unlabeled EEG datasets using techniques like masked modeling, autoregressive prediction, and instruction tuning, have demonstrated strong capabilities in learning structured spatiotemporal dependencies and in implicitly capturing the generative properties of EEG dynamics. From a modeling perspective, such architectures provide the foundation for new forms of data augmentation: masked patch modeling enables missing data reconstruction or pseudosample creation; neural tokenization combined with language-model-style decoders can support conditional EEG synthesis; and pre-trained latent representations can serve as priors for downstream generative modules. Looking ahead, these foundation models could be extended to support reconstruction-based generation, label-conditional generation, or cross-task transfer augmentation, offering more flexible and efficient solutions for data augmentation tasks.

This survey aims to guide researchers and practitioners in further advancing generative models in BCI by synthesizing current research findings and identifying future research directions, paving the way for innovative applications and breakthroughs in EEG-based artificial intelligence.

## Figures and Tables

**Table 1:** A comparison with existing reviews.

Author	Geo.	Generative Model			Tra.	EEG Tasks			
		VAE	GAN	Diffusion		MI	ER	Ext.	Hea.
Ko et al. [5]	✓	✓	✓		✓	✓	✓	✓	✓
Lashgari et al. [6]	✓	✓	✓		✓	✓	✓	✓	✓
Habashi et al. [4]		✓		✓	✓	✓	✓	✓	
Tang et al. [7]	✓	✓	✓				✓		
Carrle et al. [8]			✓						✓
Altaheri et al. [9]		✓	✓		✓	✓			
Ours	✓	✓	✓	✓	✓	✓	✓	✓	✓

Geo.: geometrical methods, VAE: Variational Autoencoder, GAN: Generative Adversarial Network, Tra.: transfer learning, MI: motor imagery, ER: emotion recognition, Ext.: external stimulation, Hea.: healthcare, ✓: indicates the method/task is addressed in the cited study.

**Table 2:** Evaluation metrics used in this review.

Category	Purpose	Common Metrics
Classification/ Detection/ Prediction/ Reconstruction	Evaluate downstream task performance	Accuracy, AUC (Area under Curve), Cohen’s kappa, F1-score, FPR (False Positive Rate), Precision, Recall, Sensitivity, Specificity, WER (Word Error Rate)
Interpretability/ Visualization	Support qualitative evaluation	ERD/ERS maps PCA (Principal Component Analysis), Spectral map, Topographic maps, t-SNE (t-distributed Stochastic Neighbor Embedding)
Task-Specific Metrics	Measure the transmission efficiency Emotion recognition	ITR (Information Transfer Rate), Valence/Arousal Scores

**Table 3:** Evaluation metrics used in this review (generative quality).

Category	Purpose	Common Metrics
Generative Quality	Evaluate the difference between generated data and real data	ABA (Amplitude Bias Analysis), FID (Fréchet Inception Distance), IS (Inception Score), I value (Mutual Information-based Index) JSD (Jensen–Shannon Divergence), KS test (Kolmogorov–Smirnov test) KL divergence (Kullback–Leibler Divergence), MAE (Mean Absolute Error) MAPE (Mean Absolute Percentage Error), MSE (Mean Squared Error), MS-SSIM (Multi-Scale Structural Similarity Index Measure), MMD (Maximum Mean Discrepancy), NMI (Normalized Mutual Information), PAD (Phase–Amplitude Distance), PCC (Pearson Correlation Coefficient), PLD (Power Level Distance), PRD (Percent Root Mean Square Difference), PSD (Power Spectral Density), RMSE (Root Mean Square Error), R value (Pearson’s Correlation Coefficient), $R^{2}$ score (Coefficient of Determination), SNR (Signal-to-Noise Ratio), SWD (Sliced Wasserstein Distance), SMAPE (Symmetric Mean Absolute Percentage Error), SD-MD (Spectral-Domain Mean Distance), WD (Wasserstein Distance)

**Table 4:** Studies that used VAEs in EEG tasks.

Study	BCI Paradigm	Dataset	Model Type	Evaluation Metrics	Results
Aznan et al., 2019 [37]	SSVEP	Private dataset [38]	VAE	Classification accuracy	+0.37
Luo et al., 2020 [39]	Emotion recognition	SEED, DEAP	sVAE	Average recognition accuracy	SVM: +3.0% DNN: +4.4%
Krishna et al., 2020 [40]	Speech recognition	Private dataset [41,42]	RNN-VAE	Classification accuracy, WER	+4.55%, −5.2% (120)
Ozan et al., 2021 [43]	Motor imagery	PhysioBank	CVAE	ERD/ERS maps, TFRs	NA
Yang et al., 2021 [44]	Motor imagery	Private dataset [44]+ BCI competition IV 2a	CVAE-GAN	IS, FID, SWD, classification accuracy	(with respect to real data) IS: −0.121, FID: +11.364, SWD: +0.067, D1: mean~+4% D2: mean~+2.5%
Bao et al., 2021 [45]	Emotion recognition	SEED, SEED IV	VAE-D2GAN	IS, FID, MMD, classification accuracy	(with respect to VAE) SEED: +0.670/ −17.197/−0.522, SEED-IV: +0.475/ −398.504/−0.678, SEED: +1.5%, SEED-IV: +3.5%
Bethge et al., 2022 [46]	Emotion recognition	SEED	EEG2Vec (CVAE)	Classification accuracy	+3%
George et al., 2022 [47]	Motor imagery	Public dataset [48,49]	AVG, RT, RF, NS, CPS, cVAE	Classification accuracy, topographic head plots, FID, t-SNE plots	(Dataset I) ACC: +0.57%, FID: ~160, (Dataset II) ACC: −0.81%, FID: ~40
Wang et al., 2022 [50]	Emotion recognition	SEED, SEED IV	MMDA-VAE	Mean accuracy	(with respect to BDAE) SEED: +9.51%, SEEDIV: +8.96%
Li et al., 2023 [51]	Epilepsy	Public dataset [52]	CR-VAE	MMD, RMSE, AUROC	MMD: 0.05, RMSE: 0.024
Zancanaro et al., 2023 [53]	Motor imagery	BCI competition IV 2a	vEEGNet	MRCP	NA
Ahmed et al., 2023 [54]	Emotion recognition	DEAP	CNN-VAE	SSIM, MAE, MSE, MAPE	1.0, 1.03 × $10^{-7}$, 1.9 × $10^{-4}$, 4.2 × $10^{-4}$
Tian et al., 2023 [55]	Emotion recognition	SEED	DEVAE-GAN	Classification accuracy	+5.00%

**Table 5:** Studies that used GANs in MI tasks.

Study	Objective	Dataset	GAN Type	Evaluation Metrics	Results
Abdelfattah et al., 2018 [60]	Improve the performance of EEG signal classification models	PhysioNet [61]	RGAN	Classification accuracy	+36.1%
Hartmann et al., 2018 [62]	Improve the stability of training	Private dataset	EEG-GAN (WGAN)	IS, FJD, ED, SWD	1.363, 9.523, −0.056, 0.078
Zhang et al., 2018 [63]	Improve the model’s classification performance	BCI competition II dataset III	cDCGAN	Classification accuracy	82.86%
Roy et al., 2020 [64]	Generate artificial EEG data for motor imagery	BCI competition IV dataset 2b	MIEEG-GAN (Bi-LSTM)	STFT spectrograms, first-/second-order characteristics, PSD	Qualitative analysis
Debie et al., 2020 [65]	Generate and classify EEG data while protecting data privacy	Graz A 2008 [66]	Privacy-preserving GAN	Classification accuracy, privacy budget, privacy guarantee	4–10%
Luo et al., 2020 [67]	Improve reconstruction quality and reduce costs	BCI competition IV dataset 2a, AO dataset, GAL dataset	WGAN (TSF-MSE loss function)	Classification accuracy	MI: +2.03%, AO: +4.1%, GAL: +4.11%
Zhang et al., 2020 [21]	Improve the model’s classification performance	BCI competition IV datasets (2b + 1)	CNN-DCGAN	Classification accuracy, kappa value, FID	2b: +12.6%, 0.677, 98.2 1: +8.7%, 0.564, 126.4
Fahimi et al, 2021 [68]	Improve the model’s classification performance	Private dataset	DCGAN	Classification accuracy	Diverted attention: +7.32% Focused attention: +5.45% IVa: +3.57%
Song et al., 2021 [69]	Improve the accuracy of cross-subject EEG signal classification	BCI competition IV dataset 2a	CS-GAN	Classification accuracy	+15.85% (3000 fake samples)
Xu et al., 2021 [70]	Improve the model’s classification performance	Private dataset	CycleGAN	Classification accuracy	+18.3%
Xie et al., 2021 [71]	Improve the model’s classification performance	BCI competition IV datasets (2a + 2b)	LGANs+ MoCNN+ Attention	Classification accuracy, R value, I value, kappa value	2a (with respect to raw data): LGAN: +8.23% Att-LAGN: +9.34% 2b (with respect to raw data): Att-LAGN: +5.64% ~6.6%
Raoof et al., 2023 [72]	Generate spatiotemporal MI EEG data	PhysioNet	cGAN + encoder + decoder	Classification accuracy, KL Divergence, KS test, PCA, t-SNE	+9.1%
Dong et al., 2023 [73]	Address the issues of EEG data scarcity or imbalance in the BCI field	Physionet	DCGAN	FFT, CWT, spectral map	Qualitative analysis
Yin et al., 2024 [74]	Improve the performance of cross-subject EEG data transfer and classification	BCI competition IV dataset 2a + OpenBMI [75]	GITGAN	Classification accuracy, F1-score, kappa value	(2a)Acc: 82.9% F1: 80.4 Kappa: 58.1 (OpenBMI) Acc: 84.0% F1: 81.4 Kappa: 58.3

**Table 6:** Studies that used GANs in emotion recognition.

Study	Objective	Dataset	GAN Type	Evaluation Metrics	Results
Luo et al., 2018 [83]	Improve the accuracies of emotion recognition models	SEED + DEAP	cWGAN	Classification accuracy, Wasserstein distance, MMD	SEED: +2.97% DEAP-Arousal: +9.15% DEAP-Valence: +20.13%
Chang et al., 2019 [84]	Recognize the emotional responses of users towards given architectural design	Private dataset [85]	GAN	Classification accuracy	+0.5%
Luo et al., 2020 [39]	Improve the accuracies of emotion recognition models	SEED + DEAP	cWGAN + sWGAN	Classification accuracy	(SEED) cWGAN + DNN: +8.3% sWGAN + DNN: +10.2% (DEAP) cWGAN + SVM: +3.5% sWGAN + SVM: +5.4%
Dong et al., 2020 [86]	Improve the accuracies of emotion recognition models	DEAP	MCLFS-GAN	Classification accuracy	(with respect to CNN+LSTM) SAP MCLFS-GAN: +14.95% LOSO MCLFS-GAN: +19.52%
Zhang et al., 2021 [87]	Address the issue of insufficient high-quality training data	SEED	MG-CWGAN	Classification accuracy, Wasserstein distance, MMD, t-SNE	SVM: +5% KNN: +2.5%
Liang et al., 2021 [88]	Extract valid and reliable features from high-dimensional EEG	MAHNOB-HCI + SEED + DEAP	EEGFuseNet	Recognition accuracy, F1-score, NMI	+7.69%, +5.07, +0.1512 (two-class) +0.0824 (three-class)
Pan et al., 2021 [89]	Solve the problem of EEG sample shortage and sample category imbalance	MAHNOB-HCI + DEAP	PSD-GAN	Recognition accuracy	Two-classification task: 6.5% and 6.71% Four-classification task: 10.92% and 14.47%
Liu et al., 2023 [90]	Generate high-quality artificial data	DEAP	CWGAN	Classification accuracy, Wasserstein distance, MMD	(Tasknet) 10.07%, 8.41, 10.76%
Qiao et al., 2024 [91]	Solve the defects in EEG with weak features and easily disturbed them	DREAMER + SEED	GAN + attention network	Recognition accuracy	(with respect to SVM) SEED: +38.14% DREAMER: +30.69%

**Table 7:** Studies that used GANs in External Stimulation.

Study	Objective	Dataset	GAN Type	Evaluation Metrics	Results
Panwar et al., 2019 [100]	Data augmentation for different cognitive events in RSVP experiments	BCIT X2	cWGAN-GP	Classifier AUC	Same subject: +0.81% (2CNN), +3.28% (3CNN) cross-subject: 3.1% (2CNN), +5.18% (3CNN)
Aznan et al., 2019 [37]	Improve the performance of SSVEP classification models	Video-Stimuli Dataset + NAO Dataset	DCGAN, WGAN	Classification accuracy	DCGAN: +3% WGAN: +2%
Panwar et al., 2020 [101]	Generate EEG signals and predict RSVP events	BCIT X2	WGAN-GP + CC-WGAN-GP	Classifier AUC, GMM log-likelihood scores	(with respect to EEGNet) +5.83%
Kunanbayev et al., 2021 [102]	Generate artificial training data for the classification of P300 in EEG	Arico et al. [103]	DCGAN + WGAN-GP	Classification accuracy, t-SNE	(Subject-specific) WGAN-GP: +0.61% (Subject-independent) WGAN-GP: +0.98%
Guo et al., 2021 [104]	Minimize the issues of insufficient diversity and poor similarity	Bi2015a	$R^{2}$WaveGAN	MS, SWD, STFT	(with respect to CC-WGAN-GP) MS: +0.2858 SWD: −0.1977
Yin et al., 2021 [105]	Generate high-quality time series prediction data	SSVEP dataset [106]	MAGAN	MSE, RMSE, MAE, MAPE, SMAPE, $R^{2}$ score	(with respect to MARNN) MSE: +0.0077 RMSE: +0.0063 MAE: −0.0455 MAPE: −3.06% SMAPE: −0.0244 $R^{2}$ score: −0.0043
Yin et al., 2021 [107]	Improve the accuracy of long-term prediction	SSVEP dataset [106]	VAECGAN	MAE, RMSE	(with respect to VAE) MAE: −0.0233 RMSE: −0.035
Xu et al., 2022 [108]	Solve class imbalance problem in RSVP tasks	Private dataset	BWGAN-GP	Classification accuracy, AUC, F1-score, Cohen’s kappa	(with respect to WGAN-GP-RSVP) Acc: +0.0391 AUC: +0.0247 F1: +0.0809 Kappa: +0.0458
Kwon et al., 2022 [109]	Convert resting-state EEG signals into specific task-state signals	Private dataset	StarGAN	Classification accuracy, ITR	(with respect to FBCCA) Acc: +3.44% ITR: +8.29 bit/min
Pan et al., 2024 [110]	Transform short-length signals into long-length artificial signals	Direction dataset + Dial dataset	TEGAN	Classification accuracy, ITR	(Direction SSVEP) Acc: +39.28% ITR: +36.36bits/min (Dial SSVEP) Acc: +37.04% ITR: +35 bits/min

**Table 9:** Studies that used GANs in other EEG tasks.

Study	Objective	Dataset	GAN Type	Evaluation Metrics	Results
Yao et al., 2018 [128]	Filter out unwanted features from EEG	UCI	CycleGAN	Classification accuracy	−66.3%
Hazra et al., 2020 [129]	Generate data to protect patient privacy	Siena Scalp EEG Database	SynSigGAN	RMSE, PRD, MAE, FD, PCC	RMSE: 0.0314 PRD: 5.985% MAE: 0.0475 FD: 0.982 PCC: 0.997
Tazrin et al., 2021 [130]	Enhance the training data to improve the model’s performance	Confused Student EEG Dataset	DCGAN	Classification accuracy	+20%
Krishna et al., 2020 [131]	Predict various types of EEG features from acoustic features	Private Dataset	RNN-GAN	RMSE	0.36
Lee et al., 2021 [132]	Fill in missing data in EEG signal sequences	Sleep-EDF Database	SIG-GAN	IS, FID, classification accuracy	(With respect to EEGGAN) IS: +0.75 FID: −5.11 Acc: 75.75%
An et al., 2022 [133]	Denoise the multi-channel EEG signal automatically	HaLT	WGAN	Correlation, RMSE	0.7771, 0.0757
Sawangjai et al., 2022 [134]	Remove ocular artifacts from EEG without EOG channels or eye-blink detection algorithms	BCI Competition IV 2b + EEG Eye Artifact Dataset + Multimodal Signal Dataset	GAN (ResNet)	PCC, RMSE, classification accuracy, F1-score	(With respect to CNN-AE) PCC: −0.011 RMSE: −3.023 (With respect to raw data) Acc: +3.1% F1: +3.7%
Abdi-Sargezeh et al., 2023 [135]	Map the sEEG to iEEG to enhance the sEEG resolution	Private Dataset	VAE-cGAN	Classification accuracy, sensitivity, specificity	(With respect to LSR) Acc: +7% (intra), +11% (inter) Sen: +7% Spec: +11%
Yin et al., 2023 [136]	Remove various physiological artifacts in EEG signals	Semi-simulated EEG/EOG + MIT-BIH arrhythmia + EEGDenoiseNet + BioSource	GAN (CNN–Transformer)	RRMSE, CC, SNR	RRMSE: −0.008 CC: +0.002 SNR: +0.313
Wickramaratne et al., 2023 [137]	Generate unique samples of non-REM sleep EEG signals	MESA	CGAN	Relative Spectral Power Visual Inspection	

**Table 10:** Studies that used diffusion models in EEG tasks.

Study	Objective	Dataset	Model Type	Evaluation Metrics	Results
Tosato et al., 2023 [149]	Address the issue of insufficient quality and quantity of EEG data	SEED	DDPM	Classification accuracy	+1.55%
Shu et al., 2023 [150]	Address the issue of data imbalance in seizure prediction	CHB-MIT + Kaggle	DiffEEG	Sensitivity, FPR, AUC	(CHB-MIT) Sens: +4.9% FPR: −0.113 AUC: +0.083 (Kaggle) Sens: +8.1% FPR: −0.047 AUC: +0.062
Aristimunha et al., 2023 [151]	Generate EEG signals of sleep stages with the correct neural oscillation	Sleep EDFx + SHHS	AE-KL+ LDM	MS-SSIM, FID, PSD	(Sleep EDFx) FID: −11.625 MS-SSIM: +0.310 (SHHS) FID: −0.768 MS-SSIM: +0.370
Sharma et al., 2023 [152]	Generate synthetic EEG embeddings to address the shortage of data	Public dataset	MEDIC	Precision, recall, F1-score, JSD score	(With respect to VAE) Precision: +0.19 Recall: +0.22 F1: +0.23
Torma et al., 2023 [153]	Generate multi-channel EEG signals with P300 components	BCI Competition III dataset II	EEGWave	IS, FID, SWD	(With respect to WGAN) IS: +0.0004 FID: −0.2623 SWD: −0.1966
Torma et al., 2023 [154]	Generate high-quality visually evoked potentials	VEPESS	DPM	SWD, IS, FID, dGMM	SWD: −72.3609 IS: +0.21 FID: −11.32 dGMM: −4988.2779
Soingern et al., 2023 [155]	Data augmentation method for motor imagery classification	BCI Competition IV 2a	WaveGrad	Classification accuracy, KL divergence	(With respect to noise) Acc: +17.17% KL: −158
Zhou et al., 2023 [156]	Improve the detection of Alzheimer’s disease	Public dataset	Diff-EEG	Accuracy, Precision, Recall	(With respect to VAE-GAN) Acc: +2.2% Precision: +3.0% Recall: +5.3%
Vetter et al., 2023 [157]	Generate realistic neuro-physiological time series	Public dataset	DDPM	Evoked potentials, PSD	Qualitative analysis
Wang et al., 2024 [158]	Diagnose MDD using EEG data	Mumtaz, 2016 [159], Arizona, 2020 [160]	DiffMDD	ACC, F1-score, recall, precision, subject-wise accuracy	(Mumtaz2016) F1-score: 91.25% ACC: 91.22% Subject-wise: 94.06% (Arizona2020) F1-score: 83.29% ACC: 83.71% Subject-wise: 85.86%
Wang et al., 2024 [161]	Protect the intellectual property for models based on EEG	DEAP	CDDPM	AUC, PCA visualization, topographic maps	AUC:0.91 (average)
Klein et al., 2024 [162]	Mitigating the class imbalance problem in ERP	Visual ERP dataset	DDPM (VP SDE)	ABA, SWD, MSE, JSD, FID, PLD, PAD, SD-MD	(With respect to baseline) FID: 0.0093 ABA: −0.001 PAD: −0.35 PLD: −0.026 SD-MD: −2.77 SWD: −0.44

## Data Availability

The study is a review article and does not involve new data.
